# Development of a two-step cultivation strategy for the production of vitamin B12 by *Bacillus megaterium*

**DOI:** 10.1186/s12934-014-0102-7

**Published:** 2014-07-15

**Authors:** Yousef Mohammed, Byong Lee, Zhen Kang, Guocheng Du

**Affiliations:** 1 The Key Laboratory of Industrial Biotechnology, Ministry of Education, Jiangnan University, Wuxi 214122, China; 2 School of Biotechnology, Jiangnan University, Wuxi 214122, China; 3 Department of Food Science/Agricultural Chemistry, McGill University, Ste-Anne-de-Bellevue H9X3V9, QC, Canada

**Keywords:** Vitamin B12, Bacillus megaterium, δ-Aminolevulinate (ALA), 5, 6-dimethylbenzimidazole (DMB)

## Abstract

**Background:**

Vitamin B12 is a fascinating molecule which acts as a co-factor in the metabolism of many organisms, especially affecting DNA synthesis and regulation, fatty acid synthesis and energy production. The synthesis of vitamin B12 is limited to a few of bacteria and archaea. Therefore, industrial microbial fermentation is used to meet annual demands worldwide of vitamin B12 and as an alternative method to the chemical synthesis which requires at least 60 steps that is uneconomical. *Bacillus megaterium* is one of vitamin B12 producers and an ideal host for many biotechnology applications and being one of the best tools for the industrial production of several enzymes. Therefore, a two-step optimization strategy was established to produce high yield of vitamin B12 by *B. megaterium* through the provision of the production requirements and the suitable conditions for the biosynthesis of vitamin B12.

**Results:**

We achieved the optimum conditions for the fermentation process of *B. megaterium* to produce high yield of vitamin B12 in a practical way based on statistical design and analysis which allowed vitamin B12 production to increase up to 759-fold (204.46 μg/l) as compared with control without parameters (0.26 μg/L). High performance liquid chromatography coupled to variable wavelength detector and mass spectrometry has been used to identify vitamin B12 forms and confirm the results.

**Conclusions:**

We developed the fermentation process of *B. megaterium* to enhance the production of vitamin B12 by providing the required supplements for the synthesis of vitamin B12 (CoCl_2_, δ-aminolevulinic acid (ALA) and 5,6-dimethylbenzimidazole (DMB)) and dividing the fermentation process into three stages. In addition, the optimum incubation times of the three fermentation stages were investigated and performed with reducing number of experimental and evaluated multiple parameters and their interactions by using statistical experimental design and analysis. All of these strategies has proven successful in enhancing the production of vitamin B12 up to 204.46 μg/l and demonstrated that *B. megaterium* could be a good candidate for the industrial production of vitamin B12.

## Background

Vitamin B12 is one of the smallest but structurally complex molecule widely recognized in the field of biological sciences [[1]]. It is a water-soluble vitamin that functions as a cofactor essential in fat biochemical reactions, protein metabolism and hemoglobin synthesis as well as DNA synthesis and regulation. It comprises of two main coenzyme forms; methylcobalamin and adenosylcobalamin [[2],[3]]. However, vitamin B12 commercially known as cyanocobalamin is a product formed by substituting adenosyl and methyl with a cyano group during the extraction process to ensure stability [[4]]. Industrial microbial fermentation is used as an alternative method to the originally established vitamin B12 chemical synthesis which requires at least 60 steps [[5]]. The natural process of vitamin B12 is synthesis by approximately 30 enzyme-mediated steps proceeding either through aerobically evident in *Pseudomonas denitrificans*, or anaerobically as witnessed in *Bacillus megaterium*, *Propionibacterium shermanii, Salmonella typhimurium* and *Lactobacillus reuteri* [[6]-[8]].

*Bacillus megaterium*, a gram-positive bacterium is one of the first established vitamin B12 producers [[8]] and has recently been embraced as a perfect model for anaerobic vitamin B12 biosynthesis thereby aiding in understanding the control and regulation of the cobalamin pathway as well as a biochemistry insight of the pathway [[9]]. Furthermore *B. megaterium* is a GRAS organism and the other features that make this strain an ideal host for the genetic modification and industrial production of vitamin B12 [[10],[11]].

Several studies have extensively assessed the genetic and biochemical aspects regarding vitamin B12 production pathway in *B. megaterium*. Moreover, attempts have been carried out in other study to engineer the bacteria using metabolic engineering tools [[12]] but the final concentration of vitamin B12 was not high (8 μg/l) probably due to the lack of understanding due to the lack of understanding the pathway of vitamin B12 and production conditions of *B. megaterium* on vitamin B12.

Establishing the production requirements and understanding of the entire vitamin B12 pathway are vital in optimizing the fermentation process to increase the productivity. Although known as a strict aerobe, *B. megaterium* can produce vitamin B12 under a very low oxygen concentrations through a complex pathway that involves at least 25 conversion steps from δ-aminolevulinate (ALA) to 5, 6-dimethylbenzimidazole (DMB) but it still requires oxygen to allow for the completion of the formation of vitamin [[13],[14]].

The aim of this research was to optimize the fermentation process of *B. megaterium* using two-step optimization strategies to produce high yields of vitamin B12. In the first strategy, the fermentation process was modified by dividing into three stages. At the first stage, the bacteria was grown aerobically to increase the biomass, followed by anaerobic growth stage for production vitamin B12 precursor and in the third stage, whole culture aeration step was used to complete vitamin B12 formation. In addition, the significance of CoCl_2,_ ALA and DMB as supplements have been investigated. In the second strategy, the optimum time for each stage in the three stages of fermentation process was investigated and optimized using Box-Behnken design combined with Response surface methodology (RSM) to investigate the most suitable time for each stage in order to achieve high yield of vitamin B12. To our knowledge this is the first report that addresses the fermentation process of *B. megaterium* to produce vitamin B12.

## Results and discussion

### Experimental design

A two-step optimization strategy was employed to increase the production of vitamin B12 by *Bacillus megaterium* through the provision of the required supplements for the synthesis of vitamin B12 (CoCl2, δ-aminolevulinic acid (ALA) and 5,6-dimethylbenzimidazole (DMB)) and the division of the fermentation process into three stages. In addition, the incubation times of the three fermentation stages were investigated and optimized in a practical way based on statistical design and analysis. All the measurements were done using High performance liquid chromatography and mass spectrometry to estimate vitamin B12 quantitatively and qualitatively.

### Confirmation of vitamin B12 analysis

In nature, there are many of vitamin B12 analogues and some of them are not active for human and have no any economic value, especially for *B. megaterium* as one of the anaerobic vitamin B12 producers reveals pseudovitamin B12 (inactive form of vitamin B12) [[9],[15]]. Therefore, there was a need to use HPLC and MS analysis to confirm the active forms of vitamin B12 (cyanocobalamin & adenosylcobalamin) unlike unreliable analysis methods such as cobalamin bioassay which is not accurate and measures all of the vitamin b12 analogues without distinction, including the inactive form of vitamin B12.

During the analyzing process, UV–vis spectra data and retention times obtained by HPLC analysis showed that *B. megaterium* produces two major forms of vitamin B12 cyanocobalamin and adenosylcobalamin due to the transformation efficiency of the cell extraction (to replace the adenosyl and methyl group to cyano group) but the yield was not high enough. By contrary, the transformation efficiency of *Propionibacterium freudenreichii* cell extraction was high in our separate experiments (data not shown). Therefore, the total concentration of vitamin B12 was used as response.

**Figure 1:**
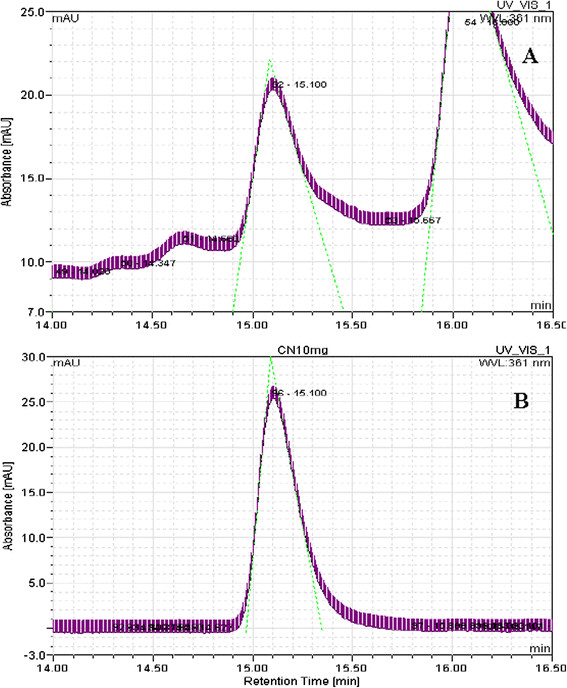
**HPLC chromatogram of cell extraction of** ***B. megaterium*** **for sample (A) and standard cyanocobalamin (B).**

**Figure 2:**
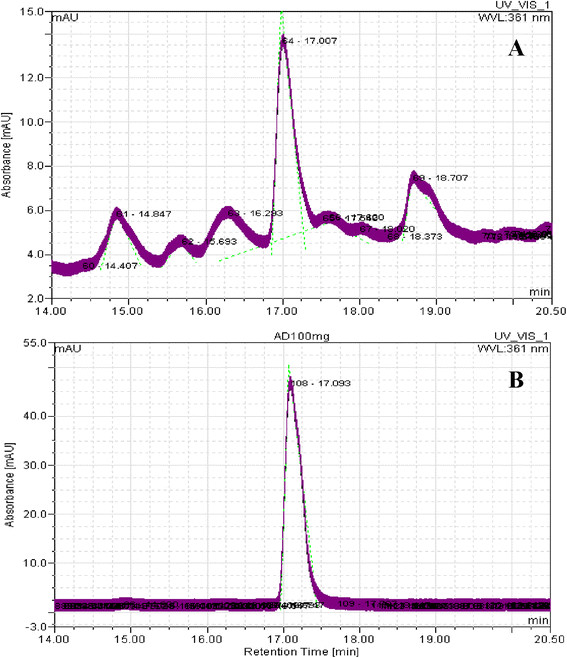
**HPLC chromatogram of cell extraction of** ***B. megaterium* for sample (A) and standard adenosylcobalamin (B).**

The cell-extracts of *B. megaterium* revealed a peak with a retention time of 15.10 min which is agreed with the cyanocobalamin standard (15.10 min) as shown in Figure 1. The cell extraction also showed a peak with a retention time 17.007 min (Figure 2A), that is in agreement with the adenosylcobalamin standard (17.093 min) in Figure 2B.

**Figure 3:**
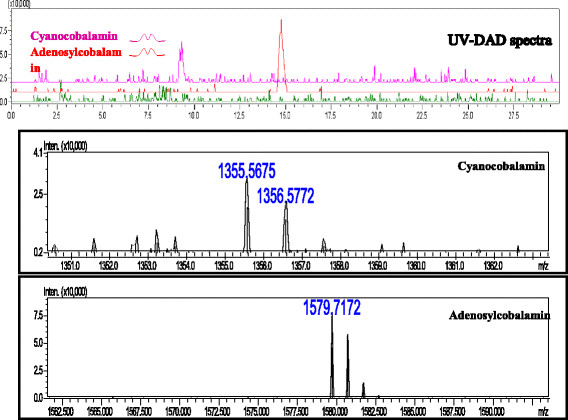
**Mass spectrometry spectrum of the corrinoids purified from** ***B. megaterium.***

The mass spectrometry data (Figure 3) showed that the mass spectra of the isolated compounds produced by *B. megaterium* are 1355.56 and 1579.71 that are very similar values of vitamin B12 analogues standard cyanocobalamin and adenosylcobalamin 1355.55 and 1579.65, respectively. Consequently, these data showed the HPLC reliability and support the results. All of the data thus demonstrated the ability of *B. megaterium* to produce adenosylcobalamin which was transformed some to cyanocobalamin during the extraction process.

### The first step of optimization

**Figure 4:**
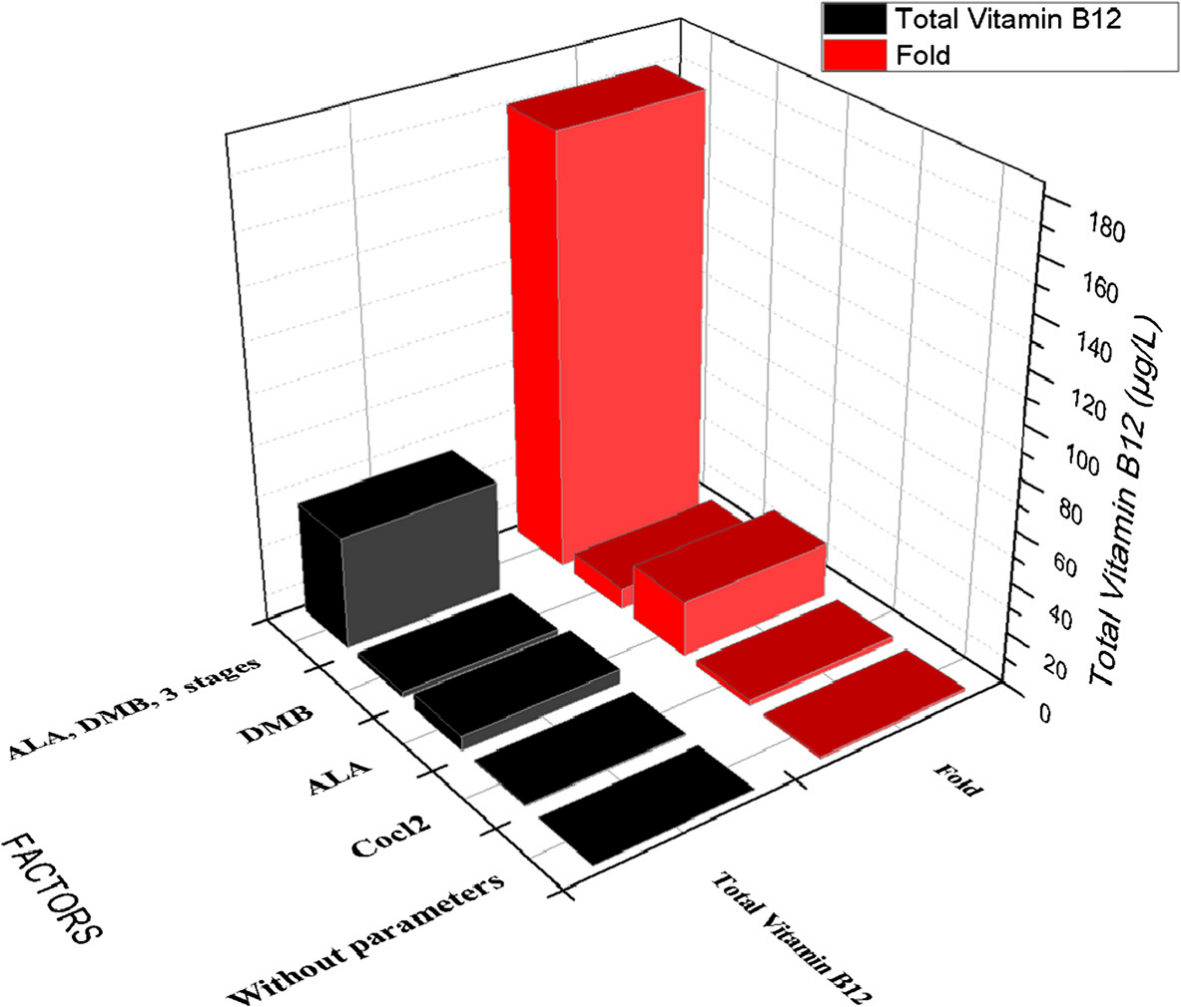
**Total vitamin B12 content from** ***B.megaterium*** **under different conditions.** All the measurements were done in triplicates with error margins of 10%.

In this part of the experiment, we evaluated the influence of CoCl_2_, ALA and DMB as supplements on vitamin B12 production by *B. megaterium*. In addition, we divided the fermentation process into three stages. This approach has proven successful. As shown in Figure 4, more than 170-fold increase of vitamin B12 was achieved by wild type of *B. megaterium* (44.38 μg/l), as compared with control without the parameter studies (0.26 μg/l) in this study and others [[16]]. Two major forms of vitamin B12, cyanocobalamin and adenosylcobalamin were present in the cell extraction of *B. megaterium c*onfirmed by UV spectra and MS data.

The starting point of this step was to optimize the first part of vitamin B12 pathway (uroporphyrinogen III synthase) and consequently increase the final product (vitamin B12). δ-aminolevulinic acid (ALA) is the key precursor of uroporphyrinogen III biosynthesis and at the beginning of vitamin B12 pathway [[7],[9]]. Many studies have been focused on increasing the productivity of ALA by the overexpression of some genes (hemA, hemL) in order to enhance the production of vitamin B12 [[16],[17]]. In our study, we added ALA (100 mg/l) directly into the culture medium and this led to more than 21-fold (5.66 μg/L) improvements in vitamin B12 (Figure 4). Although a large part of ALA flowed towards hemE along the branched pathway, several attempts were made to reduce the flow towards this pathway and also to reduce the possibility of harmful high hemE concentrations [[16]].

Regarding the final part of vitamin B12 pathway (adenosylcobinamide bioconversion), it is not completely elucidated. It required for the conversion of adenosylcobinamide to adenosylcobalamin (vitamin B12) through the replacement of the GDP group of adenosyl-GDP-cobinamide with α-ribazole which consists of the 5, 6-dimethylbenzimidazole (DMB) attached to ribose [[18]]. *B. megaterium* has proven to produce an inactive vitamin B12 (pseudovitamin-B12) instead of vitamin B12 like most of the anaerobic producers of vitamin B 12 [[9],[15]] due to the failure to provide the suitable conditions for the synthesis of DMB to complete the formation of vitamin B12. In our studies two strategies were used to optimize the last part of the anaerobic biosynthesis of vitamin B12 (adenosylcobinamide bioconversion) by adding DMB (100 mg/l) at aerobic incubation in the last phase of the fermentation that resulted more than 8-fold increase in the production of vitamin B12 (2.217 μg/L) as indicate in Figure 4.

Finally, the fermentation process of *B. megaterium* was divided into three stages. At the first stage, the cells were grown aerobically to increase the biomass and in the second stage, the bacteria were grown anaerobically to produce vitamin B12 precursor. Subsequently, vitamin B12 formation was completed by aeration of the whole culture. Taking into account, the vitamin B12 precursors ALA and DMB were added into the fermentation medium. As shown in Figure 4 the concentration of vitamin B12 was significantly increased up to 44.38 μg/l. Therefore, this factor was considered in the next step of our study.

### The second step of optimization

The experimental runs for the Box–Behnken design are shown in Table 1. The 17 runs were used to address the effects of three factors (incubation time for three stages of the fermentation process) on three responses (biomass, total concentration of vitamin B12, amount of vitamin B12 produced per g dry cells) as shown in Additional file 1.

**Table 1:** Box-Behnken matrix representing real levels of operational parameters.

	**Sample no.**	**A: Step 1 + O2** ***Hours***	**B: Step 2 -O2** ***Hours***	**C: Step 3 + O2** ***Hours***
**Run**				
1	8	18	15	18
2	14	9	15	12
3	15	9	15	12
4	1	0	6	12
5	6	18	15	6
6	11	9	6	18
7	3	0	24	12
8	5	0	15	6
9	17	9	15	12
10	13	9	15	12
11	12	9	24	18
12	10	9	24	6
13	4	18	24	12
14	9	9	6	6
15	7	0	15	18
16	16	9	15	12
17	2	18	6	12

According to the results, the second step of the optimization led to fruitful results. In addition, the ANOVA analysis for all of the responses indicated that the models used to fit the responses variables were significant (p <0.05) and adequate to represent the relationship between the responses and the independent variables. Moreover, the coefficients of determination (R^2^) of the results were more than 90%, which indicated that the results adequately represented the real relationship between the variables under consideration as shown in Additional files 2, 3, 4 and 5.

**Figure 5:**
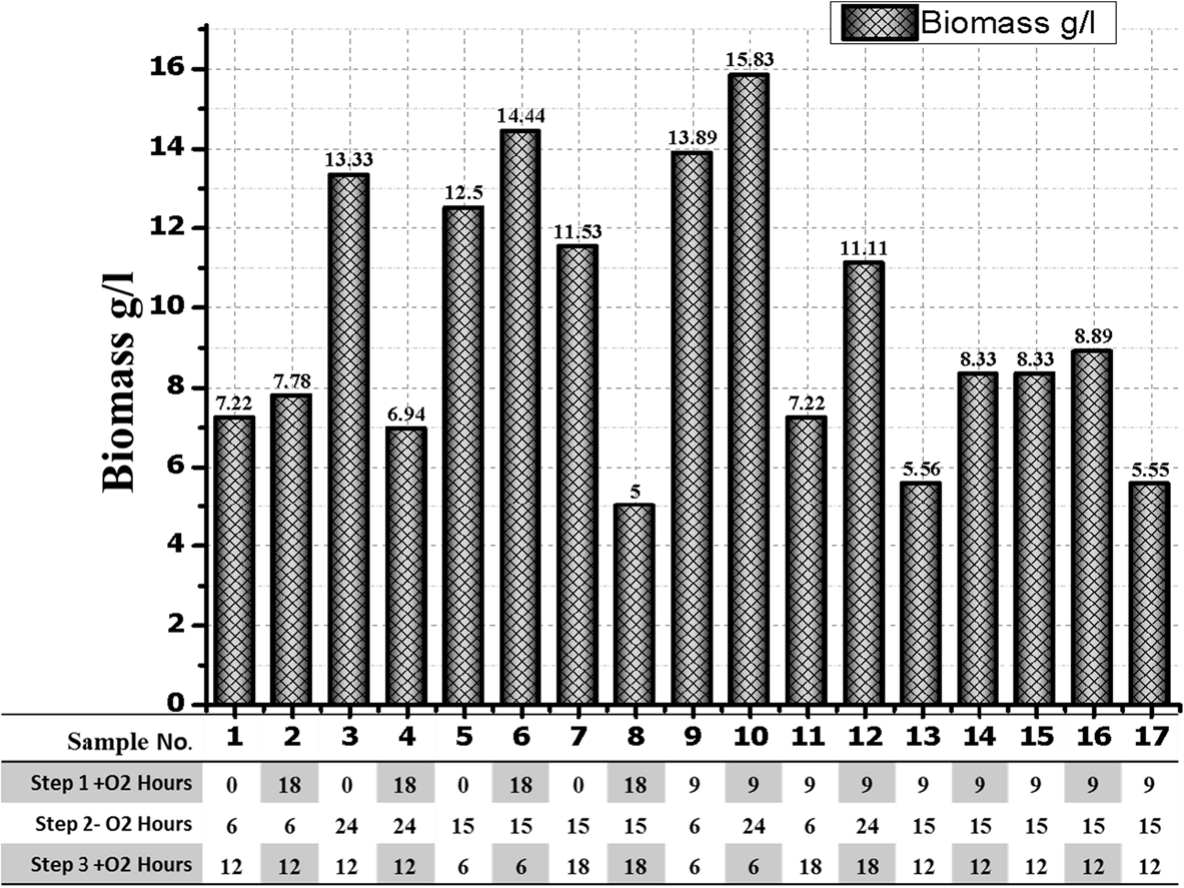
**Biomass from the second step of optimization under different growth times in third stage of fermentation with** ***B. megaterium*.** All the measurements were analyzed by ANOVA analysis with R^2^ = 94.11%, R^2^ (adjusted for df) =86.54%, Std. Dev = 1.26 and Adeq Precision =11.310.

For the biomass, the optimal bacterial biomass was 15.8 gram dry cell weight per liter culture as shown in Figure 5 and it was obtained from sample number 10 under incubation time 9 h for the first stage of the fermentation, 24 h for the second stage and 6 h for the final stage. It was clear that the highest biomass yield could be achieved by shortening the incubation time for the first and third fermentation stages when the second period of the fermentation was constant at 24 h. Furthermore, the biomass was 13.3 g DCW/l under the optimal conditions for the total vitamin B12 concentration. It has been noticed that the anaerobic condition has no substantial effects on the biomass even without the first aerobic stage. In this case, the logarithmic growth phase of the bacteria has lasted during all the second stage of the fermentation until the third stage. Moreover, we could overcome this issue by increasing the amount of the inoculation of the bacteria at the beginning of the fermentation process.

**Figure 6:**
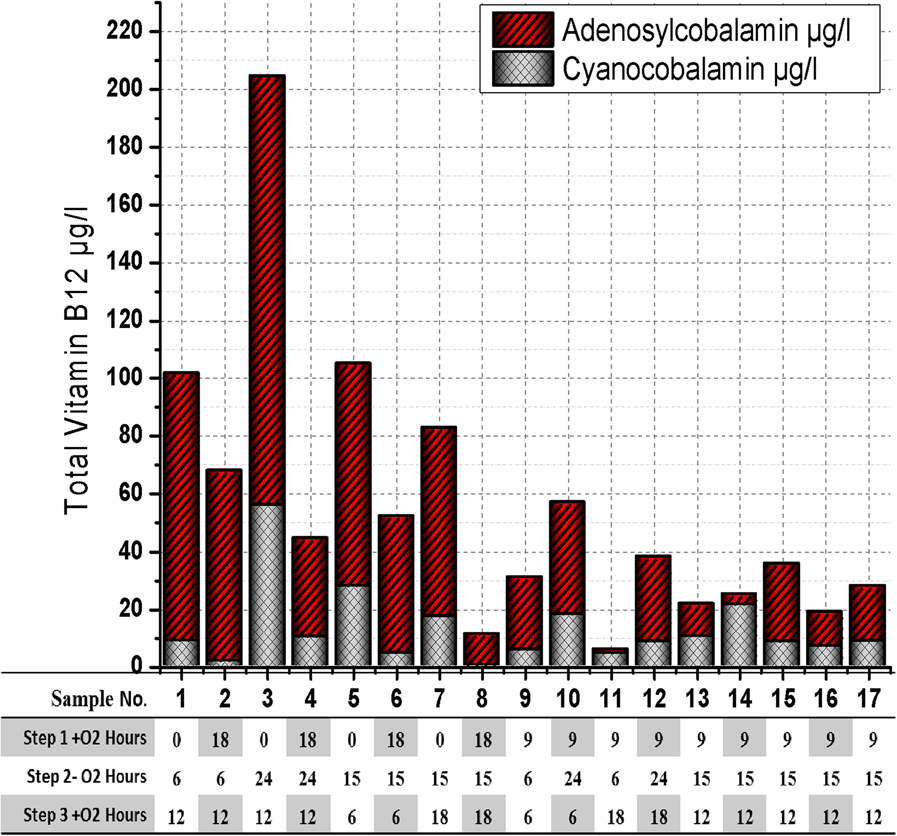
**Total vitamin B12 content from the second step of optimization under different growth times in third stage of fermentation with** ***B. megaterium*.** All vitamin B12 measurements were analyzed by ANOVA analysis with R^2^ = 97.67%, R^2^ (adjusted for df) = 94.68%, Std. Dev = 11.21 and Adeq Precision = 22.403.

In the cases of the total vitamin B12, Figure 6 indicates that the highest concentration of the total vitamin B12 per liter was achieved in sample number 3 by reducing the incubation time for the first stage to 0 h with increasing the incubation time for the second stage up to 24 h during all levels of the third stage especially at 12 h. In contrast, the increasing the incubation time for the first stage with reducing the period of the second stage led to a significant reduction in the yield of vitamin B12 as shown in Figure 6 in the sample number 8 and 11.

**Figure 7:**
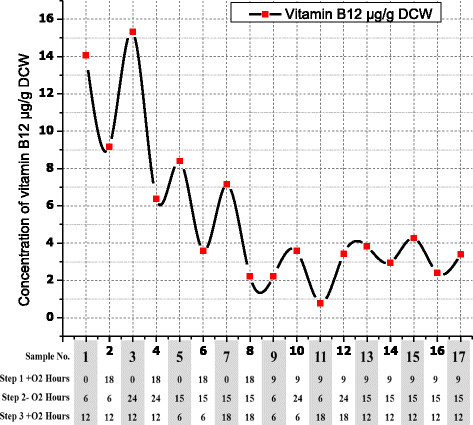
**Vitamin B12 content/g DCW from the second step of optimization under different growth times in third stage of fermentation with** ***B. megaterium*.** All vitamin B12 measurements were analyzed by ANOVA analysis with R^2^ = 97.06%, R^2^ (adjusted for df) = 93.29%, Std. Dev = 20.724, mean absolute error = 5.48 and Adeq Precision =17.175.

In relation to the final response, vitamin B12 concentrations per g DCW, the highest concentration was in sample number 3 as shown in Figure 7. A remarkable increase in the concentration of vitamin B12 per g DCW was achieved by reducing the incubation time for the first stage and increasing the time of the second stage with incubation time approximately 12 h in third stage. While the lowest concentration of vitamin B12 per g DCW was in sample number 11 due to the increase of the incubation time for the first stage and the decrease of the incubation time for the second stage.

Both of the total vitamin B12 and the concentration of vitamin B12/g DCW have shown significant agreement and compatibility. The optimal yield of vitamin B12 observed in 17 runs of the experimental design was 204.456 μg/l and 15.3342 μg/g DCW during 24 h for the second stage and 12 h for the third stage without the first stage fermentation. In general, a significant increase in the productivity was achieved when the first stage was canceled or reduced, accompanied by the increased time of the second stage up to 24 h using 6–12 h for the third stage. This is due to the fact that the synthesis of the precursors or intermediary metabolites of vitamin B 12 in the cells requires anaerobic conditions during the logarithmic phase of growth and that was provided by using the second stage (anaerobic conditions) directly at the beginning of the fermentation without resorting to use the first stage (aerobic conditions). During the optimum time for the third stage (12 h), the biomass was significantly increased along with the completion of vitamin B12 formation at the end of the logarithmic phase and throughout the stationary phase during the third stage of the fermentation process.

**Figure 8:**
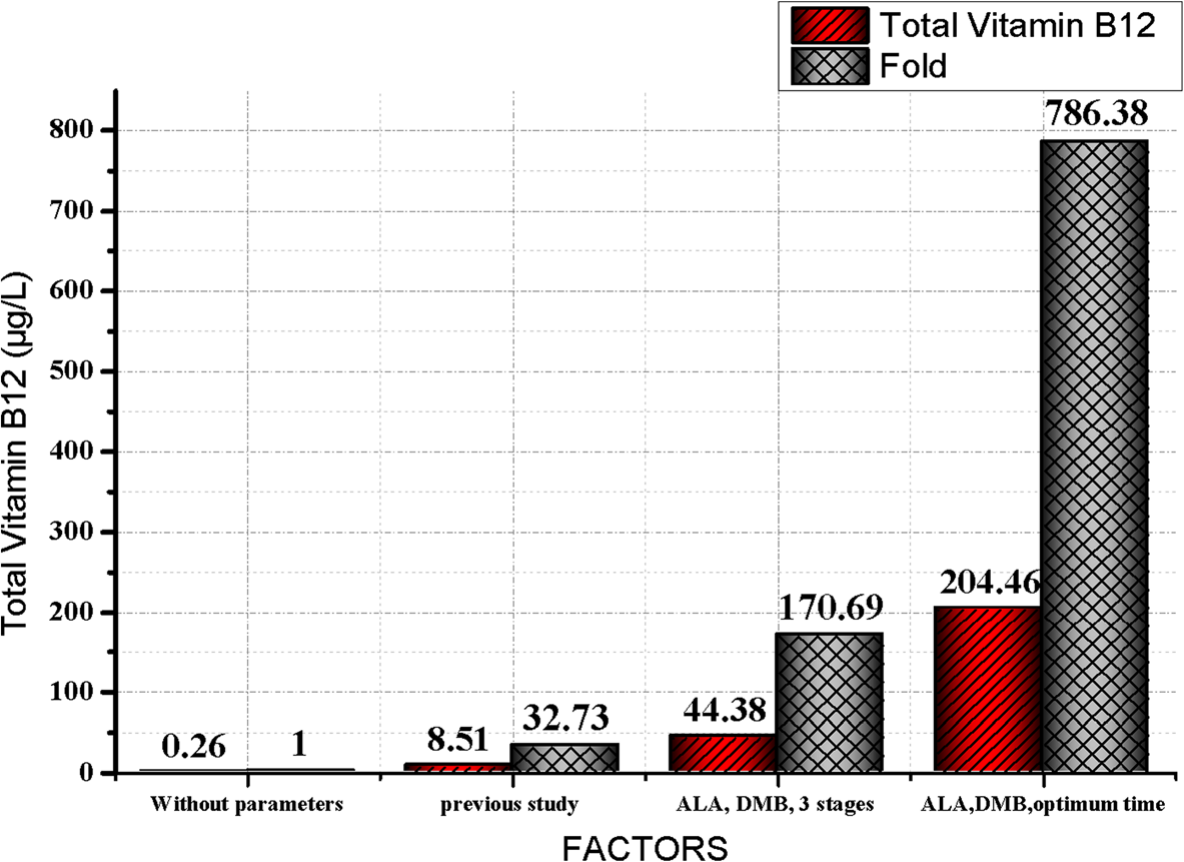
**Total vitamin B12 content in** ***B. megaterium*** **during whole optimization studies.**

Although many studies investigated the anaerobic biosynthesis of vitamin B12 in *B. megaterium* in terms of genetics and biochemical characterization [[9],[19]], optimization studies of the fermentation process have not been studied. In this study, we successfully developed new strategies for the fermentation process of *B. megaterium* to enhance the production of vitamin B12 through the sequential fermentation methods. Two major forms of vitamin B12, cyanocobalamin and adenosylcobalamin were present in the cell extraction of *B. megaterium* confirmed by UV spectra and MS data. More than 750-fold increase of vitamin B12 was achieved by wild type of *B. megaterium* (204.46 μg/l), as compared without parameters of our study (0.26 μg/l) as show in Figure 8 and more than 23-fold when we compared with the modified *B. megaterium* (8.52 μg/l) from other studies [[12],[16]].

## Conclusions

In this study, we directed our attention to increase the microbial biosynthesis of vitamin B12 by developing the fermentation process of *B. megaterium*. Two optimization steps were used to increase yield of vitamin B12 from wild type of *B. megaterium*. In the first step, the significance of CoCl_2_, ALA and DMB as supplements was investigated to increase the production of vitamin B12. In addition, we divided the fermentation process into three stages. At the first stage, the bacteria was grown aerobically to increase the biomass, followed by anaerobic growth stage for production of vitamin B12 precursor and in the third stage, whole culture aeration step was used to complete vitamin B12 formation. Using this optimization strategy, the vitamin B12 yield was enhanced up to 44.38 μg/l that is an increase of more than 170-fold as compared without the parameter studies (0.26 μg/l). In the second strategy step, the incubation times for each stage of the three stages of fermentation were investigated and optimized using Box-Behnken design combined with Response Surface Methodology (RSM) which led to increase vitamin B12 up to 759-fold as compared with control.

From our study, we deduced the three main points to improve the production of vitamin B12 by *B. megaterium*. Firstly, the synthesis of uroporphyrinogen III which derived from δ-aminolevulinic acid (ALA) could enhance ALA by some genes such as *hemA* and *hemL* or through the addition of ALA directly into the medium culture. Secondly, providing anaerobic conditions for the culture medium can enhance the synthesis of cobinamide through the oxygen-independent pathway. Thirdly, the final step of the biosynthesis of vitamin B12 includes the conversion of cobinamide to vitamin B12 through the replacement of the GDP group of adenosyl-GDP-cobinamide with α-ribazole-p which consists of DMB attached to ribose. In our studies, we stimulated *B. megaterium* to complete the synthesis of vitamin B12 by adding exogenous DMB in the culture medium under aerobic conditions in the last phase fermentation because of that bioconversion was obviously required molecular oxygen to be completed.

Further studies are underway to investigate these three points in terms of metabolic engineering through the overexpression of *hemA* and *hemL* genes form *Rhodobacter sphaeroides* 2.4.1 to increase ALA yield in addition to stimulate uroporphyrinogen III to flow towards cobalamin pathway instead of the *hemE* branched pathway by cloning *cobA* gene from *P. freudenreichii* CICC 10019. Finally, this study demonstrates that *B. megaterium* could be a good candidate for the industrial production of vitamin B12.

## Materials and methods

### Chemicals and reagents

Cyanocobalamin, adenosylcobalamin and other chemicals were purchased from Sigma. All other chemicals were of reagent grade.

### Microorganisms and media

*Bacillus megaterium* DSM319 wild-type strain was obtained from DSMZ (German Collection of Microorganisms and Cell Cultures, Braunschweig) and precultured in sterile Luria–Bertani (LB) medium which consisted of 10 g peptone, 5 g yeast extract, and 10 g NaCl. Modified TB medium was used for the fermentation and consisted of per liter, 30 g glucose, 5 g glycerol, 24 g yeast extract, and 12 g tryptone, 3 g MgSO_4_ · 7H2O, 3 g CaCO_3_, with 17 mM KH2PO_4_ and 72 mM K_2_HPO_4_. CoCl_2_ · 6H_2_O (100 mg/l), ALA (100 mg/l) and DMB (100 mg/l) at pH 6.8.

### Inoculum preparation and fermentation

After 2 ml culture was inoculated into 250 ml conical flask containing 25 ml of LB medium and incubated for 16 h at 37s with a speed of 200 rpm, 10 ml were transferred to the fermentation culture. The fermentation processes were carried out in batch mode during three stages. In the first stage, the bacteria were grown aerobically, followed by the anaerobic second stage, and the third stage was subjected to aerobic growth to complete the formation of vitamin B12. Aerobic cultivations were grown in 500 ml shake flasks containing 100 ml of modified TB at 200 rpm and 37°C. Anaerobic conditions were achieved by incubating in an anaerobic incubator at 37°C. For the optimization studies, the addition of ALA, DMB and the time for each stage of the three stages of the fermentation process was dependent on the experimental design (Table 1).

### Preparation of cell extracts

In order to analyze the yield of vitamin B12 obtained from *B. megaterium*, the bacterial cells were harvested by centrifugation (6,000 × g, 10 min, 4°C), washed twice with deionized water. The bacterial cell pellet was resuspended into 1 g/1 ml lysozyme buffer, incubated at 37°C for 30 min and followed by ultrasonic cell disruption (Sonics & Materials, Inc. 53 Church Hill Road, Newtown, CT 06470–1614 USA) for five times at 1-min interval under ice . The cell extraction was made up to a final volume of 20 ml with sodium nitrite and potassium cyanide (0.5: 0.2 g/100 ml) and then autoclaved at 120°C for 15 min. The mixture was centrifuged (8,000 × g; 10 min), and the supernatant was passed through the solid-phase extraction (SPE) column (500 mg C18 end-capped column with a 3-ml reservoir volume) previously activated with 2 ml of acetonitrile. The column was washed twice with distilled water to remove salts and other hydrophilic contaminants. Subsequently, the sample was eluted with 1 volume of 50% acetonitrile and concentrated to dry in vacuum at 30°C. The residue was dissolved in 0.5 ml of sterile distilled water and stored in the dark at 20°C until further use.

### Design of experiments

A two-step optimization strategy was employed to optimize vitamin B12 production by *B.megaterium*.

### The first step of optimization

In this step, the fermentation process was modified to consist of three stages. Moreover, the addition of CoCl_2_, ALA and DMB as supplements were studied and compared with the control bacteria without parameters according to the previous studies. Finally, the variables were investigated in order to identify which variables have significant effects on vitamin B12 biosynthesis. Four variables (CoCl_2_, ALA, DMB and the division of the fermentation process into three stages) were chosen as factors. The total concentrations of vitamin B12 after the whole fermentation were used as response in this design.

### The second step of optimization

In this step, a combination of RSM and Box–Behnken design optimizes the time of the three stages of fermentation which selected as the most significant variable by the last strategy step. The software package Design-Expert 8.0.6 (Stat Ease, Inc.,Minneapolis, USA) was used in this study. The factors were used at three different levels (−1, 0, + 1) with minimum, central and maximum values. The minimum and maximum levels of each independent variable and the experimental design are shown in Table 1.

The treatment schedule for the model is given in Table 1. Five replicates (run) at the center of the design were used to estimate the pure error and sum of squares. Seventeen experiments were randomly run as shown in Table 1. The responses obtained from the experimental design were biomass and the total vitamin B12 concentration (cyanocobalamin and adenosylcobalamin). In addition, the amount of vitamin B12 was obtained from 1 g of dry cells as the third response.

### Vitamin B12 analysis using HPLC

The corrinoids were purified from *B. megaterium* cell-extracts and analyzed using Dionex Ultimate 3000 (Thermo Scientific, Waltham, MA, USA) that comprised of an auto sampler, and an Ultimate 3000 Variable Wavelength detector (UV) set at 361 nm. Vitamin B12 concentration was analyzed at a flow rate of 0.7 ml/min. The system was completed with an Amethyst column type Acclaim C18-H (5 μm, 4.6 × 250 mm). The compositions of two mobile phases (A, B) were methanol: distilled water (60:40) and (10:90) respectively with added 0.07 mL phosphoric acid. The samples and standards were dissolved in the mobile phase and 10 μl sample volume was injected into the column at 30°C.

### Identification of Vitamin B12 by mass spectrometry

Mass spectrometric analysis of *B. megaterium* cell extraction was performed by the liquid chromatography/mass spectrometry–ion trap–time of flight (LC-MS-IT-TOF) (Shimadzu, Kyoto, Japan). Analysis parameters: Electrospray ion source; Interface voltage of −3.50 kV, Nebulizer gas flow of 1.5 L min − 1, CDL temperature of 200°C, Block heater temperature of 200°C, Detector voltage of 1.60 kV, Drying gas of 100 kpa, Ion accumulation time of 10 msec, MS range of m/z 300 to 1500, CID parameters as energy of 50%, collision gas of 100%, repeated of 10. MS data were processed with LCMS solution ver. 3.4 software (Shimadzu). Ten μl of the sample solution was introduced into the ESI ion source of mass spectrometer via the auto sampler of HPLC system with C18 column (150 × 2.0 mm; Shimadzu, Kyoto, Japan).

### Cell dry weight

Dry weight of biomass was estimated after centrifugation and drying to constant weight at 103-105°C using a vacuum oven.

### Statistical analysis

The single factor experiments were analyzed using calculated means and standard deviations by using the software Origin 8.5.1.

Analysis of variance (ANOVA) was carried out by using the software package Design-Expert 8.0.6 (Stat Ease, Inc., Minneapolis, USA) and the significance of the difference between means was determined by Duncan’s Multiple Range test (P < 0.05). Values expressed are means ± standard deviation.

## Abbreviations

ALA: δ-Aminolevulinate

DMB: 5,6-dimethylbenzimidazole

RSM: Response surface methodology

R2: Coefficients of determination

DCW: Dry cell weight

## Competing interests

The authors declare none of competing interests.

## Authors’ contributions

YM, BL, ZK and GU participated in the experimental design and established the analytical method of yield measurement. YM operated RSM software and carried out the fermentation. YM and BL participated in the statistical analysis. BL, ZK and GU carried out the validation and verification experiments. YM, BL, ZK and GU were responsible for the methods comparison and conceived of the study and helped to draft the manuscript. All authors read and approved the final manuscript.

## Additional files

## Supplementary Material

Additional file 1: **HPLC data for *Bacillus megaterium* samples (the second strategy for the optimization).** Click here for file

Additional file 2: Response surface methodology and explanation data for the second step of optimization fermentation. Click here for file

Additional file 3: ANOVA of Biomass. Click here for file

Additional file 4: ANOVA of Total vitamin B12. Click here for file

Additional file 5: ANOVA of Vitamins B12 per g CDW. Click here for file

## References

[B1] Warren MJ Raux E Schubert HL Escalante-Semerena JC The biosynthesis of adenosylcobalamin (vitamin B12) Nat Prod Rep 2002 19 390 412 10.1039/b108967f 12195810

[B2] Drennan CL Huang S Drummond JT Matthews RG Lidwig ML How a protein binds B12: a 3.0 Å X-ray structure of B12-binding domains of methionine synthase Science 1994 266 1669 1674 10.1126/science.7992050 7992050

[B3] Mancia F Keep NH Nakagawa A Leadlay PF McSweeney S Rasmussen B Secke PB Diat O Evans PR How coenzyme B12 radicals are generated: the crystal structure of methylmalonyl-coenzyme A mutase at 2 Å resolution Structure 1996 4 339 350 10.1016/S0969-2126(96)00037-8 8805541

[B4] Raux E Schubert HL Warren MJ Biosynthesis of cobalamin (vitamin B12): a bacterial conundrum Cell Mol Life Sci 2000 57 1880 1893 10.1007/PL00000670 11215515 PMC11147154

[B5] Martens J-H Barg H Warren MJ Jahn D Microbial production of vitamin B12 Appl Microbiol Biotechnol 2002 58 275 285 10.1007/s00253-001-0902-7 11935176

[B6] Taranto MP Vera JL Hugenholtz J Valdez GFD Sesma F *Lactobacillus reuteri* CRL 1098 produces cobalamin J Bacteriol 2003 185 5643 5647 10.1128/JB.185.18.5643-5647.2003 12949118 PMC193752

[B7] Kang Z Zhang J Zhou J Qi Q Du G Chen J Recent advances in microbial production of δ-aminolevulinic acid and vitamin B12 Biotechnol Adv 2012 30 1533 1542 10.1016/j.biotechadv.2012.04.003 22537876

[B8] Raux E Lanois A Warren MJ Rambach A Thermes C Cobalamin (vitamin B12) biosynthesis: identification and characterization of a *Bacillus megaterium* cobIoperon Biochem J 1998 335 159 166 9742225 10.1042/bj3350159 PMC1219764

[B9] Moore SJ Lawrence AD Biedendieck R Deery E Frank S Howard MJ Rigby SEJ Warren MJ Elucidation of the anaerobic pathway for the corrin component of cobalamin (vitamin B12) Proc Natl Acad Sci U S A 2013 110 14906 14911 10.1073/pnas.1308098110 23922391 PMC3773766

[B10] Barg H, Malten M, Jahn M, Jahn D: **Protein and Vitamin Production in**** *Bacillus Megaterium* **. In *Microbial Processes and Products, Methods in Biotechnology*, Volume 18. Edited by Barredo JL. ᅟ: ᅟ; 2005:205–223.

[B11] Vary PS Biedendieck R Fuerch T Meinhardt F Rohde M Deckwer WD Jahn D *Bacillus megaterium* from simple soil bacterium to industrial protein production host Appl Microbiol Biotechnol 2007 76 957 967 10.1007/s00253-007-1089-3 17657486

[B12] Barg H, Jahn D: *Method for the Production of Vitamin B12.* ᅟ: US Patent 20060105432 A1; 2006.

[B13] Taga ME Larsen NA Howard-Jones AR Walsh CT Walker GC BluB cannibalizes flavin to form the lower ligand of vitamin B12 Nature 2007 446 7134 449 453 10.1038/nature05611 17377583 PMC2770582

[B14] Fowler CC Brown ED Li Y Using a riboswitch sensor to examine coenzyme B12 metabolism and transport in *E. coli* Chem Biol 2010 17 756 765 10.1016/j.chembiol.2010.05.025 20659688

[B15] Mohammed Y Lee B Kang Z Du G Capability of *Lactobacillus reuteri* to produce an active form of vitamin B12 under optimized fermentation conditions JAIR 2014 2 2278 5213

[B16] Biedendieck R Malten M Barg H Bunk B Martens J-H Deery E Leech H Warren MJ Jahn D Metabolic engineering of cobalamin (vitamin B12) production in *Bacillus megaterium* Microbial Biotechnol 2010 3 24 37 10.1111/j.1751-7915.2009.00125.x PMC3815944 21255303

[B17] Murooka Y Piao Y Kiatpapan P Yamashita M Production of tetrapyrrole compounds and vitamin B12 using genetically engineering of *Propionibacterium freudenreichii.* An overview Dairy Sci Technol 2005 85 9 22 10.1051/lait:2004035

[B18] Scott AI Roessner CA Recent discoveries in the pathways to cobalamin (coenzyme B12) achieved through chemistry and biology Pure Appl Chem 2007 79 2179 2188 10.1351/pac200779122179

[B19] Moore SJ Warren MJ The anaerobic biosynthesis of vitamin B12 Biochem Soc Trans 2012 40 581 586 10.1042/BST20120066 22616870

