# Supplementary material for: Development of a two-step cultivation strategy for the production of vitamin B12 by Bacillus megaterium
Source: Microb Cell Fact. 2014 Jul 15;13:102. doi: 10.1186/s12934-014-0102-7 (PMC4105875; doi:10.1186/s12934-014-0102-7)
Supplement: Additional file 1: — HPLC data for Bacillus megaterium samples (the second strategy for the optimization). [file s12934-014-0102-7-S1.pdf]

Additional file 1. HPLC data for *Bacillus megaterium* samples (the second strategy for the optimization)

Sample No.	Run	DCW[1] *g/l*	CN[2] Area	CN[3] *µg/0.5 ml*	CN[4] *µg/l*	Ad[5] *Area*	AD[6] *µg/0.5 ml*	Ad[7] *µg/l*	Total B12[8] *µg/l*	B12[9] *µg/1g DCW*
**1**	4	7.22	0.8113	1.736	8.681	3.0433	18.575	92.8731	101.554	14.065
**2**	17	7.78	0.1634	0.341	1.748	2.1577	13.169	65.8477	67.5961	8.688
**3**	7	13.33	5.2111	11.152	55.760	4.8725	29.739	148.695	204.456	15.338
**4**	13	6.94	0.9422	2.016	10.081	1.1239	6.859	34.2983	44.3801	6.394
**5**	8	12.5	2.6056	5.576	27.880	2.5268	15.422	77.111	104.992	8.399
**6**	5	14.44	0.4171	0.893	4.463	1.5532	9.479	47.3994	51.8625	3.591
**7**	15	11.53	1.6247	3.477	17.384	2.1359	13.036	65.1818	82.5665	7.1610
**8**	1	5	0.0252	0.0539	0.269	0.3537	2.159	10.7939	11.0636	2.2126
**9**	14	13.89	0.5146	1.101	5.506	0.8303	5.0677	25.3385	30.8448	2.220
**10**	12	15.83	1.6864	3.609	18.045	1.2644	7.717	38.5873	56.6323	3.577
**11**	6	7.22	0.4095	0.876	4.382	0.0419	0.256	1.27867	5.66044	0.783
**12**	11	11.11	0.78	1.669	8.346	0.9711	5.927	29.6354	37.9817	3.418
**13**	10	5.56	0.9593	2.052	10.265	0.3744	2.285	11.4258	21.6906	3.901
**14**	2	8.33	1.9975	4.275	21.373	0.1186	0.723	3.61934	24.9932	3.000
**15**	3	8.33	0.7795	1.668	8.341	0.8919	5.444	27.2183	35.5592	4.268
**16**	16	8.89	0.6372	1.36	6.818	0.3946	2.408	12.0416	18.8598	2.121
**17**	9	5.55	0.8024	1.717	8.586	0.6321	3.858	19.2898	27.8757	5.023

[1]Dry cell weight (DCW).

[2]The peak area of the sample during the retention time of the cyanocobalamin.

[3]The concentration of the cyanocobalamin (µg) in the concentrated sample from 100 ml culture to 0.5 ml = peak area $\times$100 (standard concentration) / 46.7277 (the peak area for the standard).

[4]The concentration of the cyanocobalamin in the sample (µg) per liter = the concentration of cyanocobalamin per 0.5 ml (CN[3]) $\times$0.5 $\times$10.

[5]The peak area of the sample during the retention time of the adenosylcobalamin.

[6]The concentration of the adenosylcobalamin (µg) in the concentrated sample from 100 ml culture to 0.5 ml = peak area $\times$ 70.8 (standard concentration) / 11.6 (the peak area for the standard).

[7]The concentration of the adenosylcobalamin in the sample (µg) per liter = the concentration of adenosylcobalamin per 0.5 ml (Ad[6]) $\times$0.5 $\times$10.

[8]Total concentration of vitamin B12 (cyanocobalamin &adenosylcobalamin).

[9]Concentration of vitamin B12 per gram dry cell weight (**µg/1g DCW) =** Total vitamin B12 (B12[7]) / Dry cell weight (DCW[1]).

1. **HPLC chromatograms of standards** (cyanocobalamin (CN) and adenosylcobalamin)(Ad)

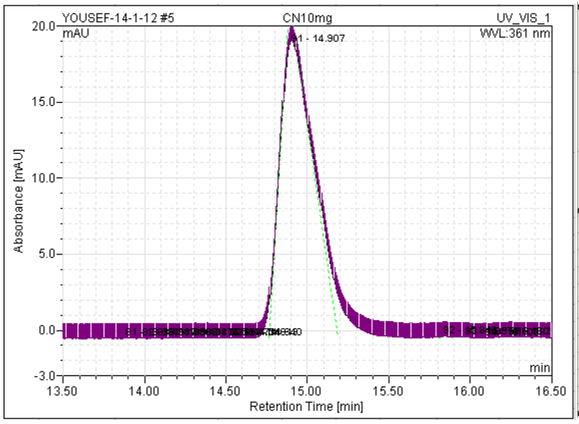

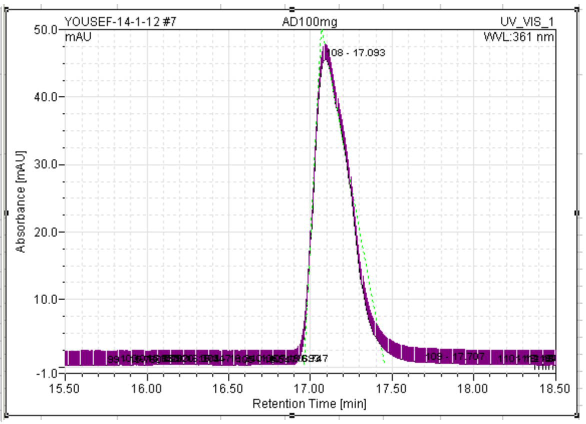

Sample	CN Retention time	CN Area	CN µg/L	Ad Retention time	Ad Area	Ad µg/L	Total B12 µg/L
CN 10mg; 100mg	14.907	5.118; 46.73	--	--	--	--	--
Ad 70.8mg	--	--	--	17.093	11.646	--	--

2. **HPLC chromatograms of sample No. 1**

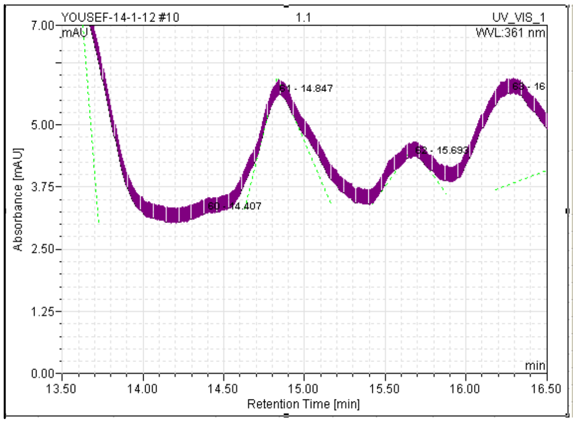

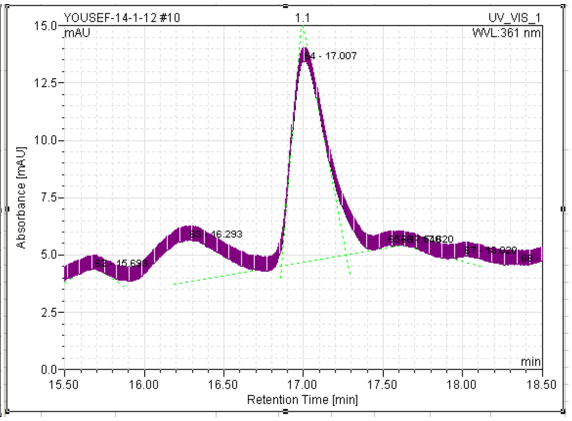

CN Retention time	CN Area	CN µg/L	Ad Retention time	Ad Area	Ad µg/L	Total B12 µg/L
14.847	0.8113	8.68	17.007	3.0433	92.87312	101.5543

3. HPLC chromatograms of sample No. 2

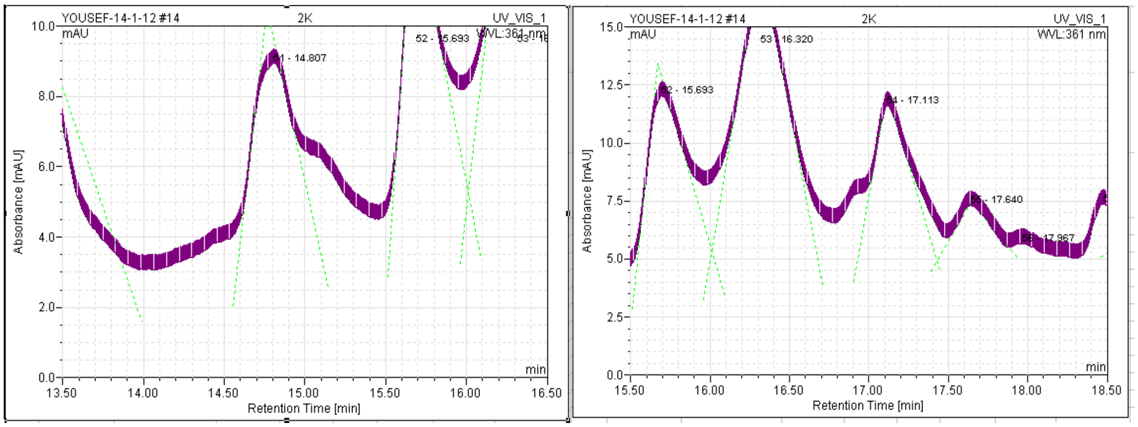

CN Retention time	CN Area	CN µg/L	Ad Retention time	Ad Area	Ad µg/L	Total B12 µg/L
14.807	0.1634	1.7484	17.113	2.1577	65.85	67.596

4. HPLC chromatograms of sample No. 3

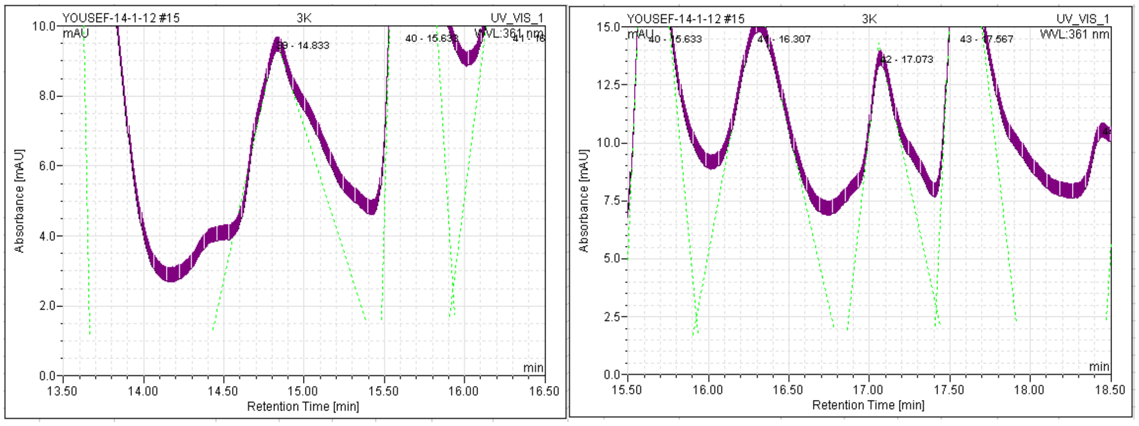

CN Retention time	CN Area	CN µg/L	Ad Retention time	Ad Area	Ad µg/L	Total B12 µg/L
14.833	5.2111	55.76	17.073	4.872	148.69	204.4555

5. HPLC chromatograms of sample No. 4

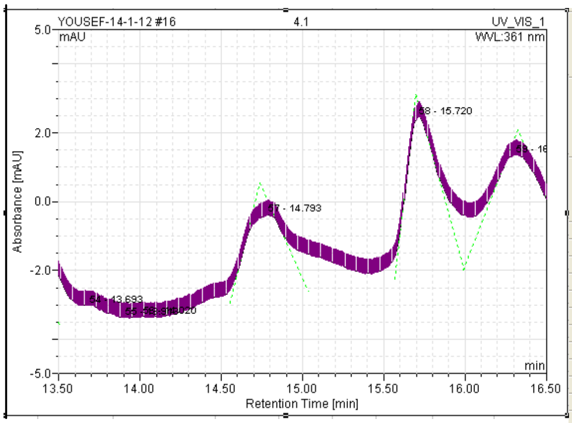

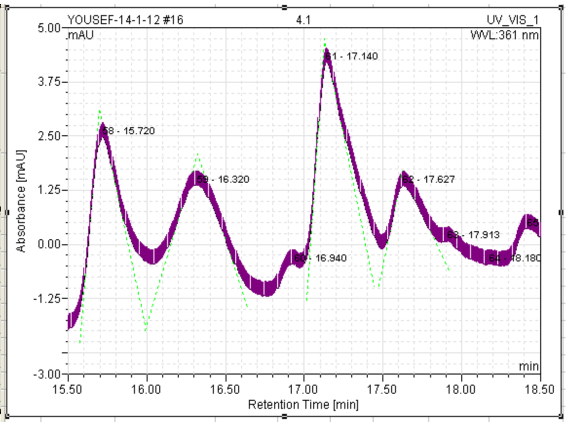

CN Retention time	CN Area	CN µg/L	Ad Retention time	Ad Area	Ad µg/L	Total B12 µg/L
14.793	0.9422	10.08181	17.140	1.1239	34.298	44.38

6. HPLC chromatograms of sample No. 5

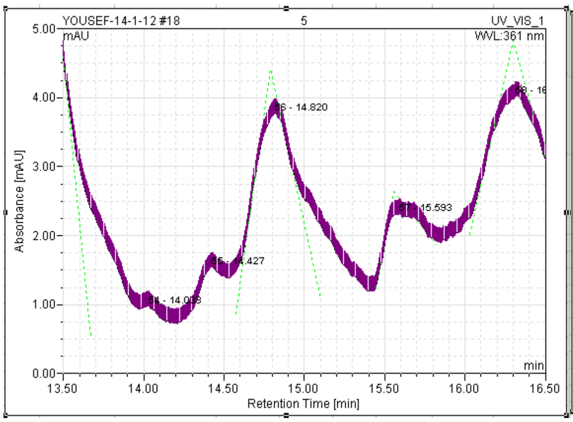

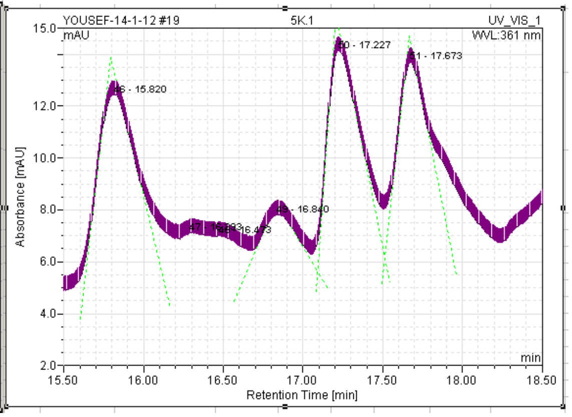

CN Retention time	CN Area	CN µ/L	Ad Retention time	Ad Area	Ad µg/L	Total B12 µg/L
14.820	2.6056	27.88	17.227	2.5268	77.11	104.99

7. HPLC chromatograms of sample No. 6

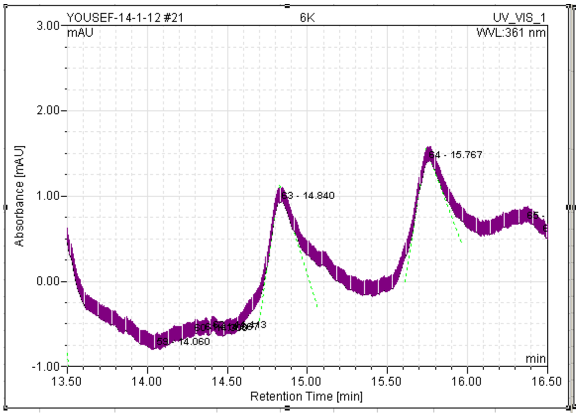

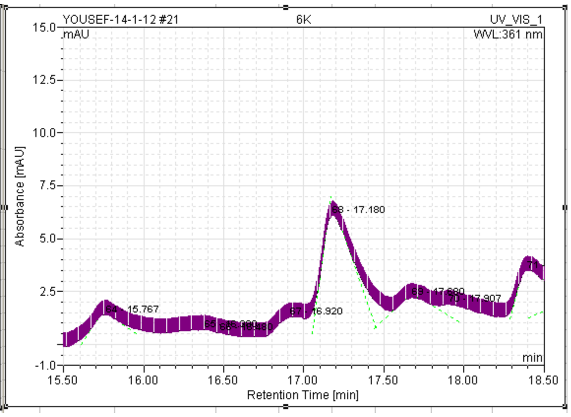

CN Retention time	CN Area	CN µ/L	Ad Retention time	Ad Area	Ad µg/L	Total B12 µg/L
14.840	0.4171	4.463	17.180	1.553	47.399	51.862

8. HPLC chromatograms of sample No. 7

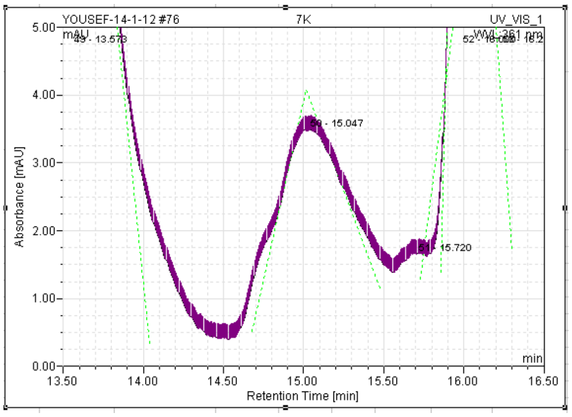

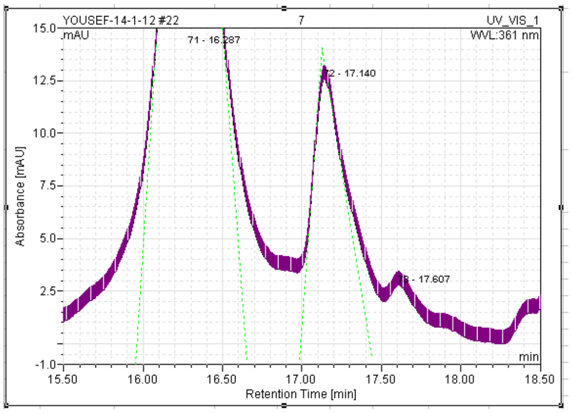

CN Retention time	CN Area	CN µ/L	Ad Retention time	Ad Area	Ad µg/L	Total B12 µg/L
15,047	1.6247	17.384	17.140	2.1359	65.181	82.566

9. HPLC chromatograms of sample No. 8

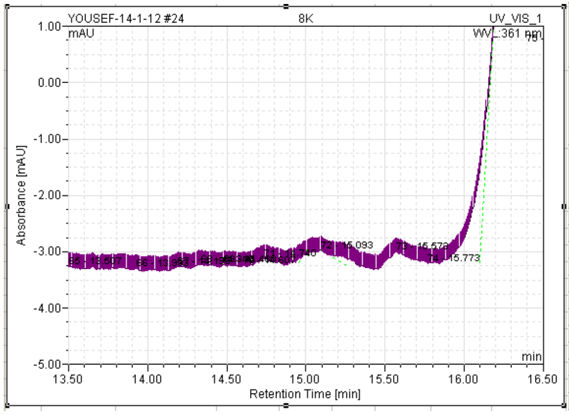

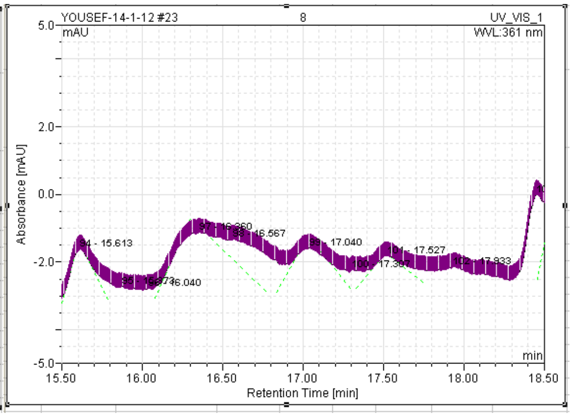

CN Retention time	CN Area	CN µ/L	Ad Retention time	Ad Area	Ad µg/L	Total B12 µg/L
15,040	0.0252	0.269	17.040	0.3537	10.79395	11.0636

10. HPLC chromatograms of sample No. 9

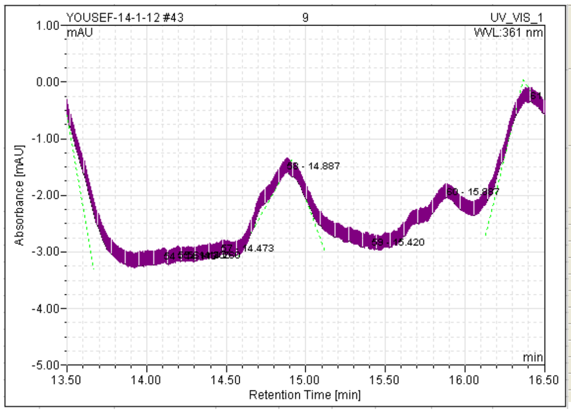

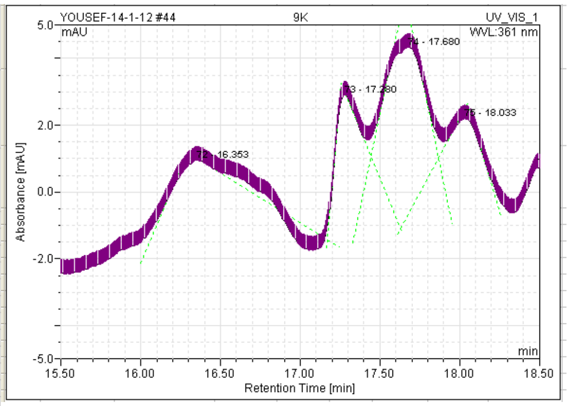

CN Retention time	CN Area	CN µ/L	Ad Retention time	Ad Area	Ad µg/L	Total B12 µg/L
14,887	0.5146	5.506	17.280	0.8303	25.338	30.844

11. HPLC chromatograms of sample No. 10

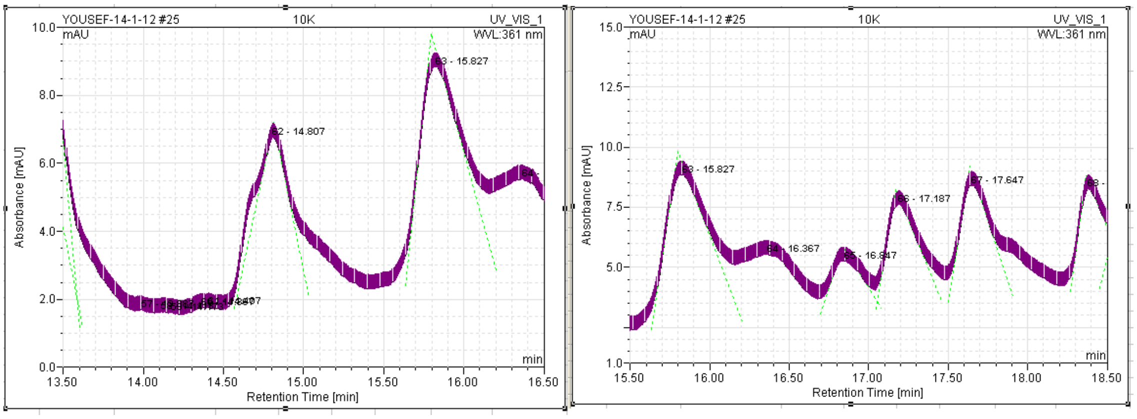

CN Retention time	CN Area	CN µ/L	Ad Retention time	Ad Area	Ad µg/L	Total B12 µg/L
14,807	1.6864	18.0449	17.187	1.2644	38.5872	56.63225

12. HPLC chromatograms of sample No. 11

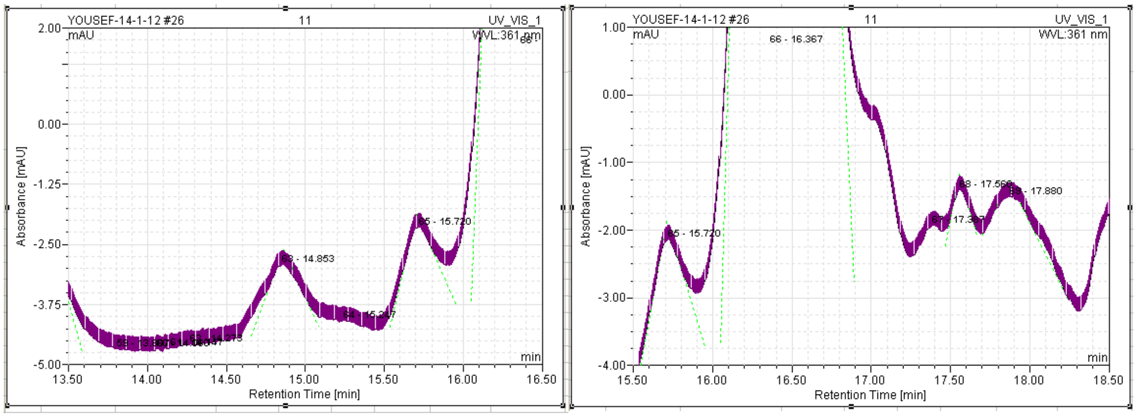

CN Retention time	CN Area	CN µ/L	Ad Retention time	Ad Area	Ad µg/L	Total B12 µg/L
14,853	0.4095	4.38176	17.387	0.0419	1.27867	5.660442

13. HPLC chromatograms of sample No. 12

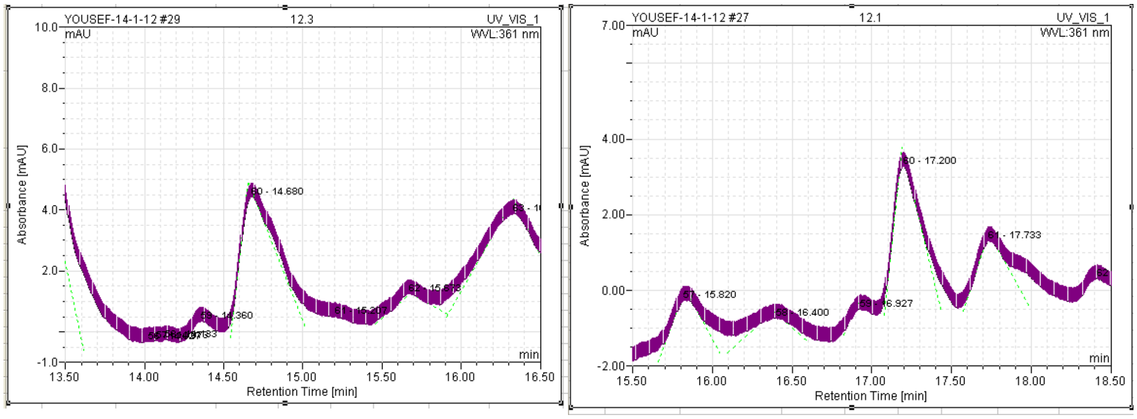

CN Retention time	CN Area	CN µ/L	Ad Retention time	Ad Area	Ad µg/L	Total B12 µg/L
14,680	0.78	8.3462	17.200	0.9711	29.6354	37.981

14. HPLC chromatograms of sample No. 13

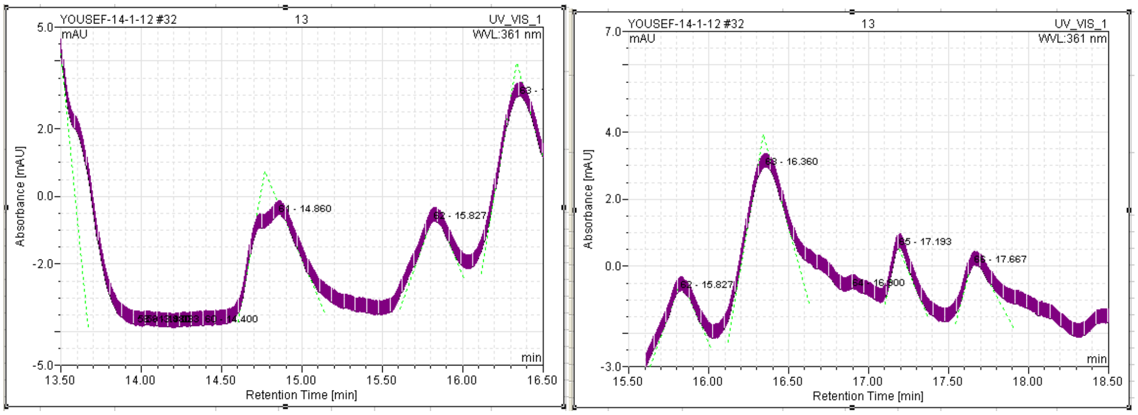

CN Retention time	CN Area	CN µ/L	Ad Retention time	Ad Area	Ad µg/L	Total B12 µg/L
14,860	0.9593	10.264	17.193	0.3744	11.4258	21.690

15. HPLC chromatograms of sample No. 14

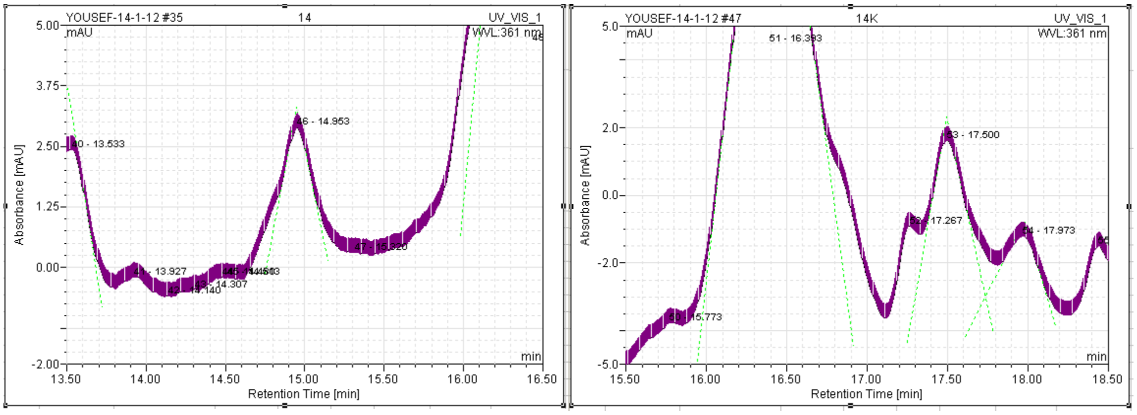

CN Retention time	CN Area	CN µ/L	Ad Retention time	Ad Area	Ad µg/L	Total B12 µg/L
14,953	1.9975	21.373	17.267	0.1186	3.61934	24.9931

16. HPLC chromatograms of sample No. 15

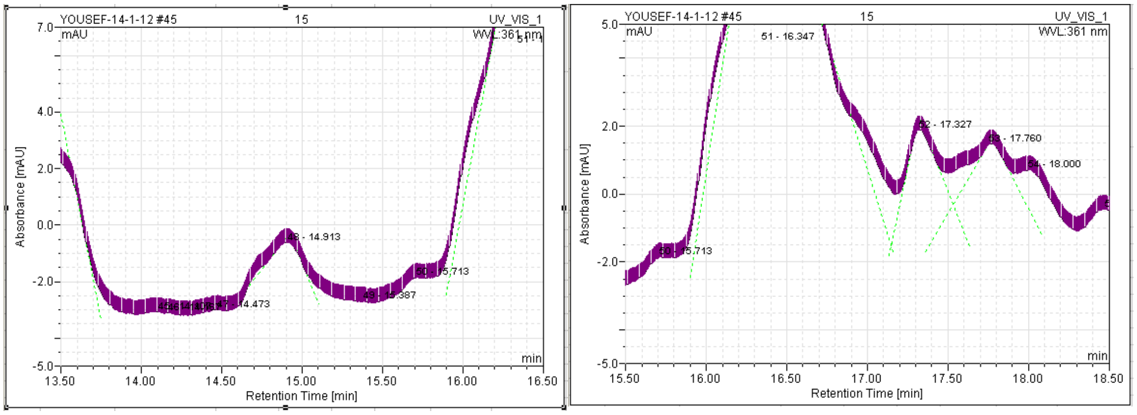

CN Retention time	CN Area	CN µ/L	Ad Retention time	Ad Area	Ad µg/L	Total B12 µg/L
14,913	0.7795	8.3408	17.327	0.8919	27.2183	35.5592

17. HPLC chromatograms of sample No. 16

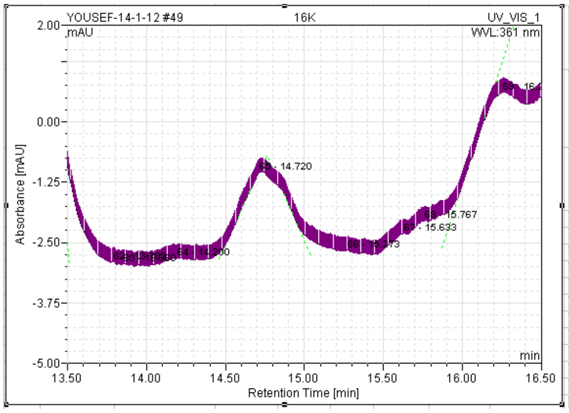

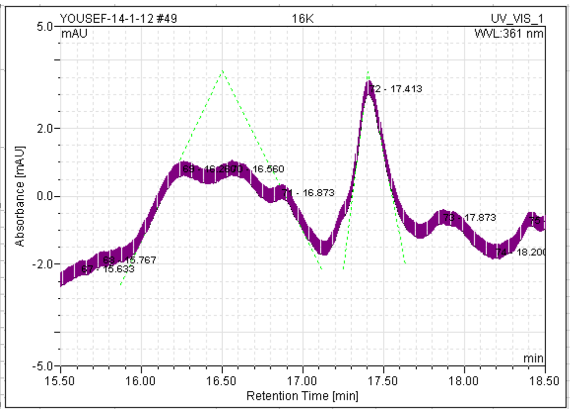

CN Retention time	CN Area	CN µ/L	Ad Retention time	Ad Area	Ad µg/L	Total B12 µg/L
14,720	0.6372	6.8182	17.413	0.3945	12.0416	18.69

18. HPLC chromatograms of sample No. 17

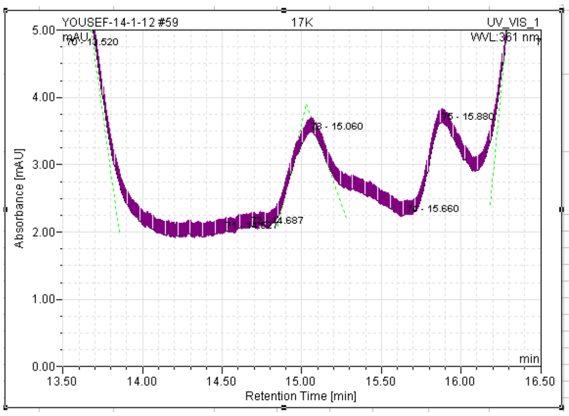

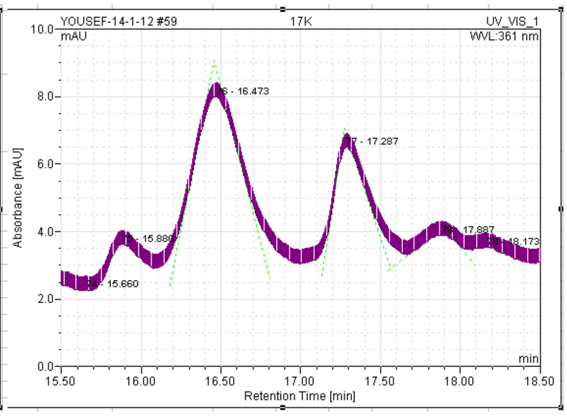

CN Retention time	CN Area	CN µ/L	Ad Retention time	Ad Area	Ad µg/L	Total B12 µg/L
15.060	0.8024	8.585	17.287	0.63209	19.289	27.8756

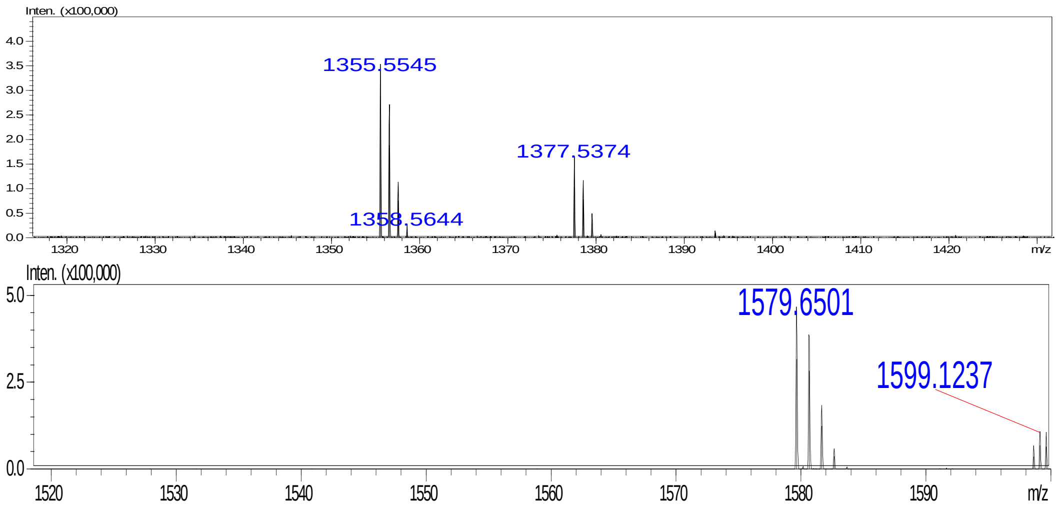

Mass spectrometry spectrum of the cyanocobalamin standard (1355.5545) and adenosylcobalamin standard (1579.6501)

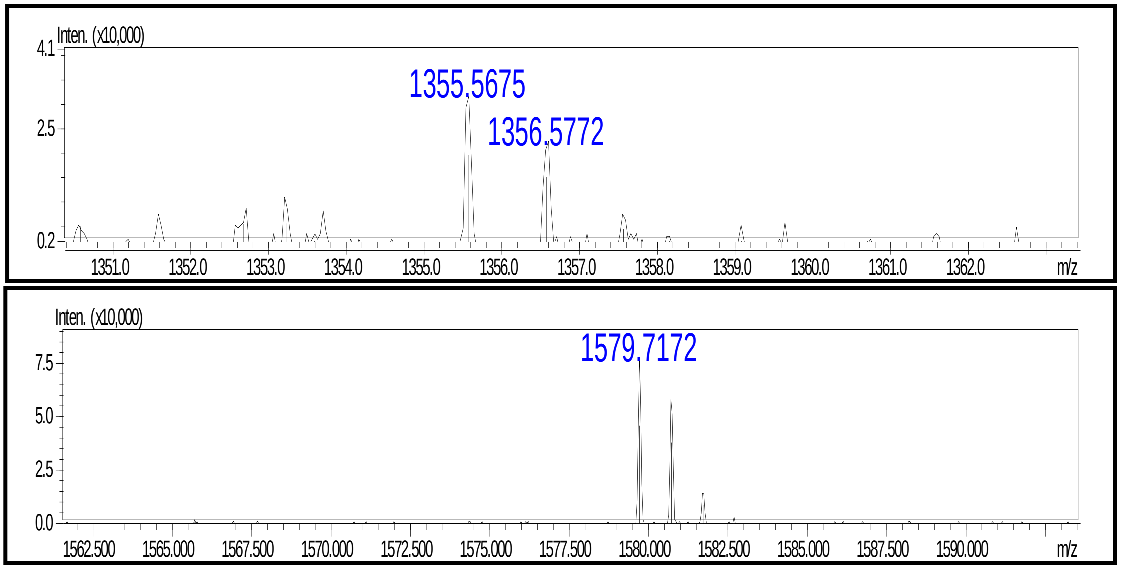

Mass spectrometry spectrum of the corrinoids purified from *B. megaterium*.
