# Supplementary material for: Development of a two-step cultivation strategy for the production of vitamin B12 by Bacillus megaterium
Source: Microb Cell Fact. 2014 Jul 15;13:102. doi: 10.1186/s12934-014-0102-7 (PMC4105875; doi:10.1186/s12934-014-0102-7)
Supplement: Additional file 2: — Response surface methodology and explanation data for the second step of optimization fermentation. [file s12934-014-0102-7-S2.pdf]

Additional file 2. Response surface methodology and explanation data for the second step of optimization fermentation

The experimental runs and results for the Box–Behnken design are shown in Table 2.1. The 17 runs were used to address the effects of three factors on three responses. The ANOVA is shown in Tables which showed the statistical significance of the biomass effects in Table 2.2, the total concentration of vitamin B12 (Table 2.3) and the amount of vitamin B12 produced per g dry cells (Table 2.4).

Table 2.1 Box-Behnken matrix representing real levels of operational parameters and observed responses.

Std	Run	A:Step 1 +O2 *Hours*	B:Step 2 -O2 *Hours*	C:Step 3 +O2 *Hours*	W *g/L*	Total B12 *µg/L*	B12 *µg/1g DCW*
8	1	18	15	18	5	11.06	2.21272
14	2	9	15	12	8.33	24.67	2.96
15	3	9	15	12	8.33	35.56	4.27
1	4	0	6	12	7.22	101.55	14.06
6	5	18	15	6	14.44	51.86	3.59
11	6	9	6	18	7.22	5.66	0.78
3	7	0	24	12	13.33	204.46	15.33
5	8	0	15	6	12.5	104.99	8.399
17	9	9	15	12	5.55	27.87	3.40
13	10	9	15	12	5.56	21.69	3.83
12	11	9	24	18	11.11	37.98	3.42
10	12	9	24	6	15.83	56.63	3.58
4	13	18	24	12	6.94	44.38	6.39
9	14	9	6	6	13.89	30.90	2.22
7	15	0	15	18	11.53	82.57	7.16
16	16	9	15	12	8.89	18.69	2.40
2	17	18	6	12	7.78	67.596	9.17

The biomass

The analysis of variance (ANOVA) in Table 2.2 showed that experimental data had correlation coefficient (R^2) of 0.9411with the calculated model which is not significant and lack of fit at P>0.05. That means the calculated model was able to explain 94.11% of the results in this case**.** The model F-value of 12.43 implies that the model is significant. There is only a 0.16% chance that a "Model F-Value" this large could occur due to noise. The values of "Prob> F" less than 0.05 indicate that model terms are significant. Six effects had P-values of less than 0.05 (Table 2.2), indicating that they were significantly different from zero at the 95% confidence level. These effects were the time of the first stage, the second stage, the third stage of the fermentation, the interaction between the time of the first stage and the second stage, the

interaction between the time of the first stage and the third stage and the quadratic effect of the third stage of the fermentation. The "Lack of Fit F-value" of 0.04 implies that the Lack of Fit is not significantly related to the pure error. There is a 98.61% chance that a "Lack of Fit F-value" this large could occur due to noise. Non-significant lack of fit is good as we want the model to fit. Adequate precision measures the signal to noise ratio and a ratio greater than 4 is desirable. An adequate precision of 11.31 indicates an adequate signal. This model can be used to navigate the design space.

Table 2.2 Analysis of variance (ANOVA) for the biomass results (R^2=94.11%, R^2 (adjusted for df) =86.54%, Std. Dev = 1.26, mean absolute error= 9.62, Adeq Precision =11.310).

Source	Sum of Squares	df	Mean Square	F Value	p-value Prob >F	
**Model**	**177.23782**	**9**	**19.693092**	**12.427144**	**0.0016**	**Significant**
**A-Step 1 +O_2**	**13.563368**	**1**	**13.563368**	**8.5590383**	**0.0222**	
**B-Step 2 -O_2**	**15.432099**	**1**	**15.432099**	**9.7382836**	**0.0168**	
**C-Step 3 +O_2**	**59.435282**	**1**	**59.435282**	**37.506086**	**0.0005**	
**AB**	**12.056327**	**1**	**12.056327**	**7.6080341**	**0.0282**	
**AC**	**17.944637**	**1**	**17.944637**	**11.323798**	**0.012**	
**BC**	**0.945216**	**1**	**0.945216**	**0.5964699**	**0.4652**	
**A^2**	**0.1218831**	**1**	**0.1218831**	**0.0769132**	**0.7895**	
**B^2**	**7.2917174**	**1**	**7.2917174**	**4.601371**	**0.0691**	
**C^2**	**47.664931**	**1**	**47.664931**	**30.078515**	**0.0009**	
**Residual**	**11.092785**	**7**	**1.5846836**			
**Lack of Fit**	**0.3520448**	**3**	**0.1173483**	**0.0437021**	**0.9861**	**not significant**
**Pure Error**	**10.740741**	**4**	**2.6851852**			
**Cor total**	**188.33061**	**16**				

The total amount of vitamin B12 per liter culture

The coefficient of determination (R^2) was 0.9767 with the calculated model with no significant lack of fit at p>0.05. That means 97.67 of the variabilities of this response were explained by the model and only 3.67% was as a result of chance. The results obtained from the ANOVA analysis for the concentration of vitamin B12 (Table 2.3) indicated that the model used to fit the response variable was significant ($p < 0.05$) and adequate to represent the relationship between the responses and the independent variables. There are seven effects with P-values of less than 0.05 as given in Table 2.3. These effects were the time of the first stage, the second

stage and the third stage of the fermentation, the interaction between the time of the first stage and the second and the quadratic effect of the first, the second and the third stage of the fermentation. Adequate precision value (12.625) measures the signal to- noise ratio, and a ratio greater than 4 is generally desirable.

Table 2.3 Analysis of variance (ANOVA) for the total vitamin B12 concentration (R^2=97.67%, R^2 (adjusted for df) =94.68%, Std. Dev = 11.21, Adeq Precision =22.403).

Source	Sum of Squares	df	Mean Square	F Value	p-value Prob > F	
**Model**	**36890.7**	**9**	**4098.967**	**32.64353**	**< 0.0001**	**significant**
**A-Step 1 +O2**	**12693.46**	**1**	**12693.46**	**101.0888**	**< 0.0001**	
**B-Step 2 -O2**	**2371.417**	**1**	**2371.417**	**18.88559**	**0.0034**	
**C-Step 3 +O2**	**1434.237**	**1**	**1434.237**	**11.42204**	**0.0118**	
**AB**	**3976.402**	**1**	**3976.402**	**31.66744**	**0.0008**	
**AC**	**84.39919**	**1**	**84.39919**	**0.672142**	**0.4393**	
**BC**	**10.86076**	**1**	**10.86076**	**0.086493**	**0.7772**	
**A^2**	**12440.53**	**1**	**12440.53**	**99.07439**	**< 0.0001**	
**B^2**	**2533.465**	**1**	**2533.465**	**20.17612**	**0.0028**	
**C^2**	**1266.873**	**1**	**1266.873**	**10.08918**	**0.0156**	
**Residual**	**878.9727**	**7**	**125.5675**			
**Lack of Fit**	**707.2826**	**3**	**235.7609**	**5.492709**	**0.0667**	**not significant**
**Pure Error**	**171.6901**	**4**	**42.92252**			
**Cor. total**	**37769.67**	**16**				

Vitamin B12 concentration µg/g DCW

In relation to the final response, vitamin B12 concentrations per g dry cell and the coefficient of determination (R^2) of the model were 0.9706, which indicated that the model adequately represented the real relationship between the variables under consideration. A R^2 value of 0.8738 means that 97.06% of the variability was explained by the model. The analysis of variance (Table 2.4) evaluated that the presented model is significant ($p <0.05$). A model F-value of 25.70 implies that the model is significant. There is only a 0.01% chance that a "Model F-Value" this large could occur due to noise. The "Lack of Fit" F-value of 3.76 implies that there is insignificant lack of fit. The "Lack of Fit" (Prob> F) value of 0.1166 implies that there is only 11.66 % chance that the “Lack of Fit" F-value could occur due to noise. The adequate precision value of 17.175 indicates an adequate signal and it can be used to navigate the design space.

Table 2.4 Analysis of variance (ANOVA) for the concentration of vitamin B12 per 1g DCW results (R^2=97.06%, R^2 (adjusted for df) =93.29%, Std. Dev = 20.724, mean absolute error=5.48, Adeq Precision =17.175).

Source	Sum of Squares	df	Mean Square	F Value	p-value Prob > F	
**Model**	**267.5808**	**9**	**29.7312**	**25.69966**	**0.0001**	**Significant**
**A-Step 1 +O2**	**69.55827**	**1**	**69.55827**	**60.12618**	**0.0001**	
**B-Step 2 -O2**	**0.766431**	**1**	**0.766431**	**0.662503**	**0.4425**	
**C-Step 3 +O2**	**2.220058**	**1**	**2.220058**	**1.919019**	**0.2085**	
**AB**	**4.112485**	**1**	**4.112485**	**3.554833**	**0.1014**	
**AC**	**0.004958**	**1**	**0.004958**	**0.004286**	**0.9496**	
**BC**	**0.41138**	**1**	**0.41138**	**0.355597**	**0.5697**	
**A^2**	**120.7049**	**1**	**120.7049**	**104.3373**	**< 0.0001**	
**B^2**	**26.6097**	**1**	**26.6097**	**23.00143**	**0.002**	
**C^2**	**48.24113**	**1**	**48.24113**	**41.69965**	**0.0003**	
**Residual**	**8.0981**	**7**	**1.156871**			
**Lack of Fit**	**5.97857**	**3**	**1.992857**	**3.76094**	**0.1166**	**not significant**
**Pure Error**	**2.11953**	**4**	**0.529883**			
**Cor. total**	**275.6789**	**16**				

Multiple regression analysis on the experimental data was obtained after the analysis of ANOVA; results are shown for all of the responses (Y), considering (A) time for the first stage of the fermentation, (B) time of the second stage, and (C) time for the third stage.

Biomass = +7.33-1.30 A +1.39 B – 2.73 C – 1.74 AB – 2.12 AC + 0.49 BC + 0.17A2 +1.32 B2 +3.36 C2

Total vitamin B12 = + 25.61 – 39.83 A + 17.22 B – 13.39 C – 31.53 AB – 4.59 AC + 1.65 BC + 54.36 A^2 + 24.53 B^2 – 17.35 C^2

Vitamin B12 per 1g DCW = +3.37 – 2.95 A + 0.31 B – 0.53 C – 1.01 A B – 0.035 AC + 0.32 BC + 5.35 A^2 + 2.51 B^2 – 3.38 C^2

3.3.3.4 The interaction between the factors (3D response surface)

In order to see the effects of the independent variables and their mutual interaction on all of the responses, the three-dimensional (3-D) response surfaces were obtained after analysis. The results showed different shapes which indicated variation in the combined effect of independent variables on the responses. The three-dimensional (3D) plots were generated by keeping one variable constant at the optimum point and varying the others within the experimental range. The resulting response surfaces have shown the effect of each stage of the fermentation on the biomass, total concentration of vitamin B12 and amount of vitamin B12 in per g DCW.

Figure 2.1A-C show the interaction between the times of the fermentation stages on the biomass. As shown in Figure 2.1A the increase of the time of the first stage was significant and caused an increase in the biomass only during short period of the second stage from 6 h to 15 h.

Figure 2.1B indicates that the highest biomass yield could be achieved by short fermentation time in the first and third stage when the second period of the fermentation was constant at 24 h. However, the biomass was decreased due to the increased time of the first stage, accompanied by increased time of the third stage.

The interaction between the time of the second step and the third step of the fermentation shown in Figure 2.1C reveals that the biomass showed a slow increase when the period of the third step was short, while the second step time was insignificant in this case.

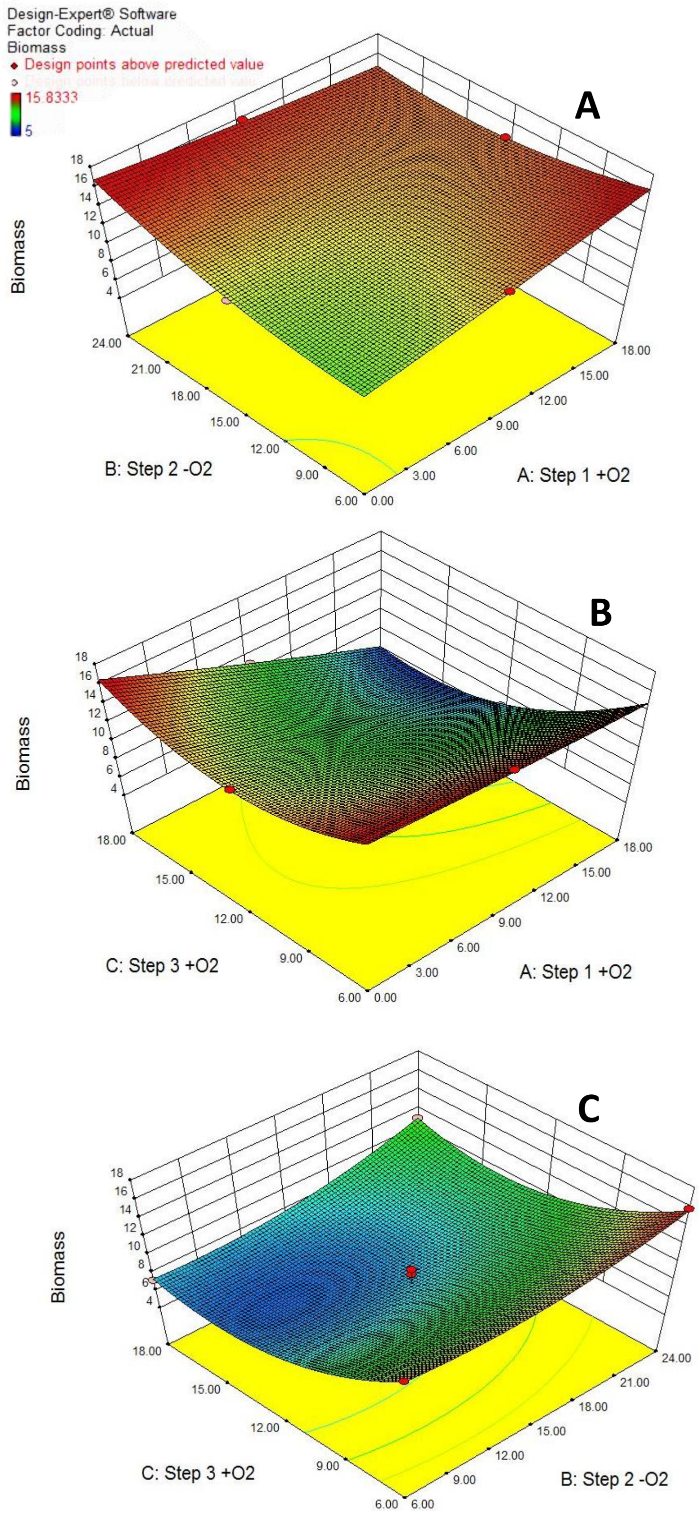

Figure 2.1 The interaction between the different factors on the biomass.

Figure 2.2A-C show the contour plots of the effect of the incubation times for the fermentation stages on total vitamin B12 concentration. Figure 2.2A shows the effect of the time of the first and the second stage during the optimum time for the third stage (12 h). The total concentration of vitamin B12 was increased when the incubation time for the second stage was increased up to 24 h with the decrease of the first stage time down to 0 h.

Figure 2.2B depicts the production of vitamin B12 under interaction of time of the first and the third stage of fermentation when the second stage time was at the optimum level (24 h). Reduction of the time of the first stage was caused a large increase in the yield of vitamin B12 during all levels of the third stage.

Figure 2.2C shows the effect of the incubation times for the second and the third stage of the fermentation on the total vitamin B12 concentration at the optimum level of the first stage. The time of the second stage was significant and caused an increase in the total vitamin B12 during all levels of the third stage, because the third stage is insignificant in this case.

Figure 2.2D-F describes the interaction between the times of the fermentation stages on the concentration of vitamin B12 per g DCW. The interaction between the first stage and the second stage of the fermentation during the optimum time for the third stage was showed in Figure 2.2D. The decrease of the time in the first stage was significant and caused an increase in the concentration of vitamin B12 combined with the increase of the second stage up to 24 h.

Figure 2.2E indicates that the higher concentration of vitamin B12 was achieved by reducing the first stage fermentation to 0 h during all levels of the third stage especially at 12 h at the fixed second period of the fermentation at 24 h.

Finally, Figure 2.2F shows the concentration of vitamin B12 under interaction of time in the second and the third stage of the fermentation when time of the first stage at the optimum level was at 0 h. The amount of vitamin B12 increased when the incubation time for third stage was approximately 12 h during all levels of the second stage.

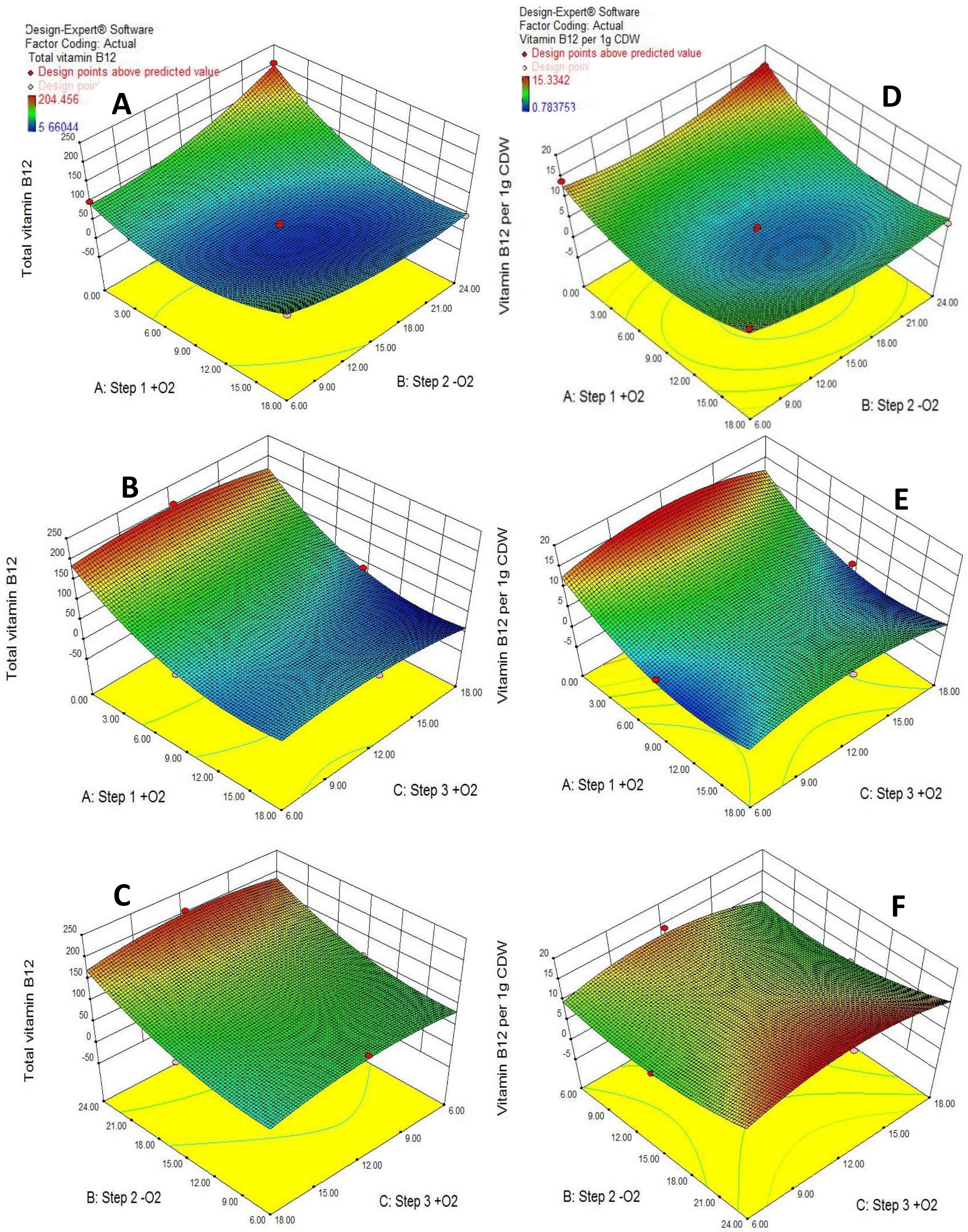

Figure 2.2 The interaction between the different factors on total vitamin B12 in (A, B, C) and vitamin B12 per g DCW in (D, E, F).
